# Monoterpenoid Indole Alkaloids from Inadequately Dried Leaves of *Alstonia scholaris*

**DOI:** 10.1007/s13659-015-0066-2

**Published:** 2015-08-18

**Authors:** Xu-Jie Qin, Yun-Li Zhao, Chang-Wei Song, Bei Wang, Ying-Ying Chen, Lu Liu, Qiong Li, Dan Li, Ya-Ping Liu, Xiao-Dong Luo

**Affiliations:** State Key Laboratory of Phytochemistry and Plant Resources in West China, Kunming Institute of Botany, Chinese Academy of Sciences, Kunming, 650201 People’s Republic of China; University of Chinese Academy of Sciences, Beijing, 100049 People’s Republic of China

**Keywords:** *Alstonia scholaris*, Inadequately dried leaves, Indole alkaloids, Alstoniascholarine, Bioactivities

## Abstract

Six new indole alkaloids, named alstoniascholarines L–Q (**1**–**6**), together with nineteen known analogues were isolated from the inadequately dried leaves of *Alstonia scholaris*. Their structures were elucidated on the basis of extensive analysis of spectroscopic data and by comparison of their physical and spectroscopic data with the literature values. In addition, the new alkaloids were tested for their cytotoxic and neurite outgrowth-promoting activities.

**Electronic supplementary material:**

The online version of this article (doi:10.1007/s13659-015-0066-2) contains supplementary material, which is available to authorized users.

## Introduction

Plants of the genus *Alstonia* (Apocynaceae), which are usually shrubs or trees, grow mainly in the tropical regions of Africa and Asia. *A. scholaris* has been historically used to treat chronic respiratory diseases in ‘dai’ ethnopharmacy in Yunnan province, People’s Republic of China. Previous phytochemical and pharmacological studies on this species afforded a number of structurally diverse indole alkaloids [1–5] with various bioactivities, such as antineoplastic [6], antibacterial [7], anti-inflammatory and analgesic effects [8], and broncho-vasodilatory [9] activities. The leaves of *A. scholaris* are usually collected and dried by exposed to sunshine in an open yard. However, some leaves of *A. scholaris* might not be dried in time because of rainy days in Pu’er city. Then, the green color of these leaves might fade in couples of day even they were died finally. Whether these leaves can be used for medical raw material is still unknown, which encouraged us to carried out HPLC analysis of total alkaloids from the inadequately dried leaves of *A. scholaris.* The results indicated that picrinine, the major bioactive compound was decreased remarkably and more peaks were appeared in the HPLC fingerprint profile of inadequately dried leaves (see Supplementary data). Correspondingly, the anti-tussive efficacy reduced significantly (Table 1). In our continuing efforts to search for structurally interesting and bioactive indoles of this plant [10–15], the inadequately dried leaves of *A. scholaris* were investigated. As a result, six new indole alkaloids, alstoniascholarines L–Q (**1**–**6**), along with nineteen known analogues were isolated. We report herein the isolation, structural elucidation and bioactivities of alkaloids.

**Table 1:** Effect of the different alkaloid extracts on ammonia-induced cough in mice

Group	Dose (mg/kg)	Frequency of cough	Inhibition (%)
Control		31.3 ± 6.5	–
Codeine phosphate	30	7.0 ± 2.5**	77.6
Alkaloids from the dried leaves	20	13.1 ± 4.8**	58.0
	10	17.0 ± 5.6**	45.6
Alkaloids from the inadequately dried leaves	20	19.4 ± 3.9**	38.0
	10	22.8 ± 7.1*	27.2

Values expressed as mean ± SEM (*n* = 10), ^*^ *P* < 0.05 and ^**^ *P* < 0.01 for comparison of treated groups with control

## Results and Discussion

Alkaloid **1** was obtained as a white amorphous powder. The HREIMS spectrum showed a quasi-molecular ion peak at *m*/*z* 356.1366 [M]^+^ (calcd for C_19_H_20_N_2_O_5_, 356.1372) from which, in conjunction with the ^13^C NMR data, the molecular formula was determined to be C_19_H_20_N_2_O_5,_ requiring 11 indices of hydrogen deficiency. The UV absorption maxima 204, 240, and 298 nm suggested the presence of an indole chromophore, while the IR spectrum of **1** indicated the presence of indolic amino (3440 cm^−1^), ester carbonyl (1748 cm^−1^), and aromatic ring (1612 and 1485 cm^−1^) functionalities, respectively. The ^13^C NMR and DEPT spectra (Table 2) for **1** revealed 19 carbon signals, including characteristic signals due to an indole ring [*δ*_C_ 109.2 (s, C-2), 51.2 (s, C-7), 136.7 (s, C-8), 124.3 (d, C-9), 121.1 (d, C-10), 130.2 (d, C-11), 110.3 (d, C-12), and 148.7 (s, C-13)], two ester carbonyls (*δ*_C_ 178.1 and 178.8), one oxygen-bearing quaternary carbon (*δ*_C_ 92.8), four methines (*δ*_C_ 68.9, 51.2, 49.1, and 32.3), three methylenes (*δ*_C_ 45.7, 44.2, and 20.7), and one methyl group (*δ*_C_ 17.5).

**Table 2:** ^1^H and ^13^C NMR spectroscopic data for alkaloids **1**–**3** in CD_3_OD

Position	**1** ^a^		**2** ^a^		**3** ^b^	
	*δ*_C_	*δ*_H_ (*J*, Hz)	*δ*_C_	*δ*_H_ (*J*, Hz)	*δ*_C_	*δ*_H_ (*J*, Hz)
2	109.2		109.0		84.5	
3	51.2	3.63 br d (5.7)	51.4	3.64 br d (5.8)	68.5	3.66 br d (3.1)
5	178.1		178.1		150.7	8.81 d (4.8)
6a	44.2	3.73 d (18.0)	44.0	3.78 d (18.0)	118.4	7.49 d (4.8)
6b		2.77 d (18.0)		2.75 d (18.0)		
7	51.2		51.2		nd^c^	
8	136.7		136.6		128.1	
9	124.3	7.20 d (7.5)	124.2	7.20 d (7.5)	129.2	9.19 d (8.6)
10	121.1	6.79 t (7.6)	121.0	6.80 t (7.5)	128.0	7.69 t (8.4)
11	130.2	7.08 t (7.5)	130.2	7.08 t (7.7)	131.0	7.79 t (8.2)
12	110.3	6.62 d (7.8)	110.3	6.62 d (7.8)	129.7	8.06 d (8.3)
13	148.7		148.7		150.1	
14a	20.7	1.92 ddd (8.0, 6.0, 2.0)	21.5	2.00 ddd (8.1, 6.1, 2.0)	26.0	2.22 d (13.8)
14b		1.77 dd (14.6, 4.3)		1.77 dd (14.5, 4.3)		1.98 dt (13.8)
15	32.3	2.83 m	34.3	2.62 m	42.5	3.11 m
16	49.1	3.23 d (11.5)	48.6	3.28 d (11.5)	55.7	3.36 d (7.9)
17	178.8		178.7		176.5	
18	17.5	1.20 d (6.5)	17.4	1.20 d (6.5)	17.5	1.26 d (6.5)
19	68.9	3.72 q (6.5)	71.3	3.78 q (6.5)	70.4	3.81 q (6.5)
20	92.8		92.1		92.0	
21a	45.7	3.06 d (13.3)	44.6	3.24 d (13.4)	54.2	2.94 d (12.8)
21b		2.68 d (13.3)		2.73 d (13.4)		2.79 d (12.8)
*N*-Me					46.2	2.65 s

^a^Recorded at 400 and 100 MHz

^b^Recorded at 600 and 150 MHz

^c^Not detected

**Scheme 1:**
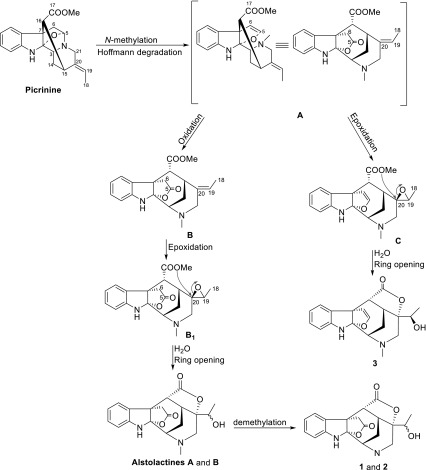
Putative biosynthetic pathway of alkaloids **1**–**3** from picrinine

**Fig. 1:**
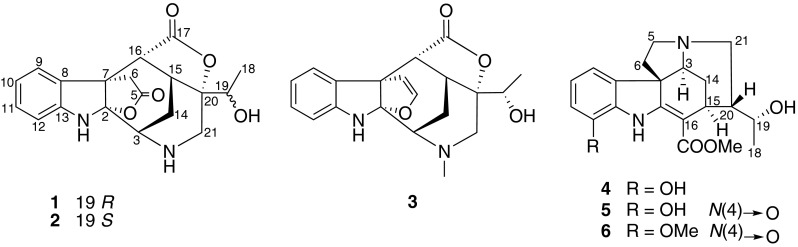
Structures of alkaloids **1**–**6**

**Fig. 2:**
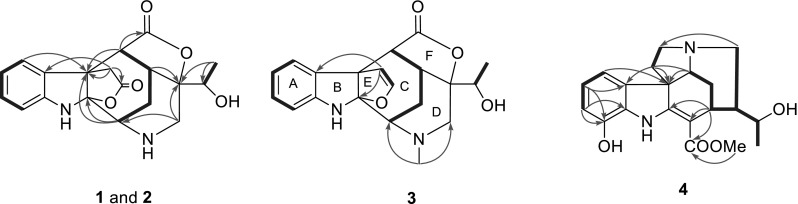
Key ^1^H-^1^H COSY () and HMBC () correlations for **1**–**4**

**Fig. 3:**
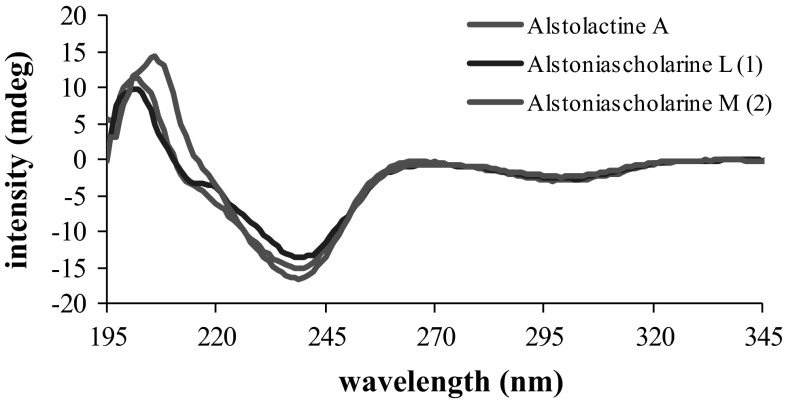
Experimental CD spectra of alstolactine A (*red*), **1** (*blue*) and **2** (*green*). (Color figure online)

The ^1^H–^1^H COSY spectrum of **1** disclosed the presence of three structural fragments, **a** (C-9–C-12), **b** (C-3–C-14–C-15–C-16), and **c** (C-18–C-19), as shown in Fig. 2. The position of functional groups and the skeleton of alkaloid **1** were assigned by its HMBC data. In the H MBC spectrum, the correlations of *δ*_H_ 3.73 and 2.77 (both d, *J* = 18.0 Hz, H_2_-6) with *δ*_C_ 109.2 (s, C-2), 178.1 (s, C-5) and 51.2 (s, C-7), indicated the presence of a five-membered lactone ring E which was formed by the connection of C-5 and C-2 via an oxygen atom. Besides, the HMBC correlations of H-16 with C-7 and of *δ*_H_ 3.63 (1H, br d, *J* = 5.7 Hz, H-3) with C-2 suggested the formation of a six-membered ring C. The relative downfield shifts of *δ*_C_ 51.2 (d, C-3) and 45.7 (t, C-21) required that they both be connected to a nitrogen atom. Likewise, the HMBC correlations of *δ*_H_ 2.68 (1H, d, *J* = 13.3 Hz, H-21b) with *δ*_C_ 32.3 (d, C-15), 92.8 (s, C-20) and C-3, and of *δ*_H_ 2.83 (1H, m, H-15) with C-20 established the occurrence of another six-membered ring. Moreover, considering the last one degree of unsaturation in **1**, another ring should be constructed. The key HMBC correlations of H-16 with C-17 and C-20 revealed that another five-membered lactone ring was present between C-17 and C-20. Finally, a linkage of C-18/19/20 was deduced from HMBC correlations of Me-18 (*δ*_H_ 1.20, d, *J* = 6.5 Hz) with *δ*_C_ 68.9 (d, C-19) and C-20. On the basis of the aforementioned information, the planar structure of **1** was elucidated as an monoterpenoid indole alkaloid with a rare 6/5/6/6/5/5 fused ring system. The stereochemistry was then considered. In the ROESY spectrum, the correlations of H-3/H_2_-14, H_2_-14/H-15, and H-15/H-16 were observed, indicating that they should be placed on the same side. However, it still could not determine the configuration for such complicated ring system. According to the similarities between the NMR data of **1** and the recently reported bio-relationship (Scheme 1) of alstolactines A–C [14], the configuration of **1** was assigned as 2*R*, 3*S*, 7*R*, 15*R*, 16*R*, 19*R*, 20*S*. This was further confirmed by the comparison of its CD spectrum with that of alstolactine A (Fig. 3). It is worthy to note that alstolactines A–C were also isolated from the leaves of *A. scholaris* and their absolute configurations were determined by the X-ray diffraction. On the basis of the above results, the structure of alkaloid **1** was established to be as shown in Fig. 1 and named alstoniascholarine L.

Alkaloid **2** was shown to have the same molecular formula of C_19_H_20_N_2_O_5_ as that of **1** based on its HREIMS spectrum (*m*/*z* 356.1358 [M]^+^, calcd for C_19_H_20_N_2_O_5_, 356.1372). The ^1^H and ^13^C NMR spectral data (Table 2) of **2** were almost identical with those of **1**, except for the carbon signals of C-15, C-19, and C-21, which indicated that **2** could be a 19-epimer of **1**. Following a similar analysis of the ^1^H, ^13^C and 2D NMR spectroscopic data as described before, the gross structure of alkaloid **2** was the same as that of **1**. The well matched CD curves of alkaloids **1** and **2** (Fig. 3) proposed the absolute configurations of **1** and **2**. The absolute configurations of 2*R*, 3*S*, 7*R*, 15*R*, 16*R*, 20*S* were those commonly accepted from biogenetic point of view. However, the configuration of C-19 was assigned as 19*R* rather than 19*S* based on its similar NMR data with that of alstolactine B [14]. Therefore, the structure of **2** was characterized as shown in Fig. 1 and named alstoniascholarine M.

Alkaloid **3** was determined to have the molecular formula of C_20_H_22_N_2_O_4_ from an HREIMS ion peak at *m*/*z* 354.1577 ([M]^+^). The ^1^H and ^13^C NMR spectroscopic data (Table 2) of **3** showed its structure resembling that of alstolactine A. The main difference between them was the presence of two olefinic carbon signals (*δ*_C_ 118.4 and 150.7) in alkaloid **3** and the absence of the lactone (*δ*_C_ 177.2) and methylene (*δ*_C_ 43.8) groups in **2**. The HMBC correlation of H-5 (*δ*_H_ 7.49, d, *J* = 4.8 Hz) with C-7 (*δ*_C_ 84.5) and a ^1^H-^1^H COSY correlation between H-5 (*δ*_H_ 7.49) and H-6 (*δ*_H_ 8.81) allowed the location of the double bond was at C-5 and C-6. Other parts of **3** were identical to those of alstolactine A, as supported by its HSQC, HMBC, and COSY experimental data. The configuration of C-19 was determined to be *R* based on the chemical shifts of C-19 (*δ*_C_ 70.4) and C-20 (*δ*_C_ 92.0), which were in agreement to those of alstolactine A (*δ*_C_ 69.2 and 92.1) rather than those of alstolactine B (*δ*_C_ 71.7 and 91.4). The other configurations of **3** were assigned to be the same as those of **1** based on their same biogenetic pathway (Scheme 1) and similar NMR data. Accordingly, the structure of **3** was elucidated as shown in Fig. 1 and named alstoniascholarine N.

Biosynthetically, the three related alkaloids might be derived from the precursor picrinine, which was also isolated as a main chemical constituent in this experiment. A plausible biogenetic pathway for **1**–**3** suggested that a Hoffmann degradation led to the formation of a dihydrofuran intermediate **A**. Further oxidation would yield a lactone derivative **B**. Then, epoxidation at C-20–C-19 double bond, followed by ring opening could produce another lactonic F-ring. Subsequently, alkaloids **1** and **2** would finally be obtained via demethylation of the methyl *N*(4)-Me of alstolactines **A** and **B**. Coincidently, alkaloid **3** might also derive from intermediate **A** without oxidation and demethylation.

**Fig. 4:**
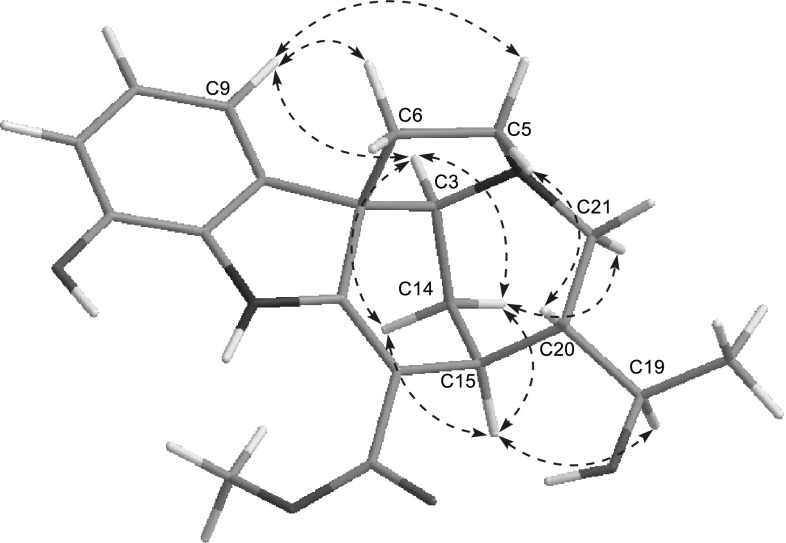
Key ROESY () correlations for alkaloids **4**–**6**

Alkaloid **4**, isolated as a white amorphous powder, had the molecular formula C_20_H_24_N_2_O_4_, as established by its HRESIMS data (*m*/z 357.1810, [M + H]^+^, calcd for C_20_H_25_N_2_O_4_ 357.1810), which indicated 10 degrees of unsaturation. Its IR spectrum displayed characteristic absorptions attributing to amino/hydroxyl (3417 cm^−1^), double bond (1678 cm^−1^), and aromatic ring (1620, 1440 cm^−1^) functionalities. The ^1^H NMR spectrum (Table 3) showed the presence of a 1, 2, 3-trisubstituted aromatic moiety due to the signals of three contiguous aromatic hydrogens (*δ*_H_ 6.89, d, *J* = 7.5 Hz, H-9; 7.14, t, *J* = 7.8 Hz, H-10; 6.82, d, *J* = 8.1 Hz, H-11) and two methyls (*δ*_H_ 3.79 and 1.32). The ^13^C NMR spectrum (Table 3) displayed a total of 20 carbon signals, which were classified as two methyls, four methylenes, seven methines, and seven quaternary carbons, respectively. The presence of conjugated ester functionality was supported by the observed carbon signals at *δ*_C_ 168.4 and 51.7, while the signals due to the two olefinic quaternary carbons at *δ*_C_ 165.6 (C-2) and 105.3 (C-16) are consistent with the presence of a *β*-anilinoacrylate moiety [8]. One downfield signal at *δ*_C_ 69.0 was associated with the presence of oxymethine. The COSY and HMQC data (Figs. 2, 3) disclosed the following partial structures, viz., *N*CH_2_CH_2_, *N*CHCH_2_CHCHCH_2_, and CHCHCH_3_, corresponding to the C-5–C-6, C-3–C-14–C-15–C-20-C–21, and C-20–C-19–C-18 fragments, respectively. The above NMR data and the partial structures from the COSY spectrum indicated a similarity *N*(4)-demethylalstogustine (**25**) except for the observed difference in the aromatic region. The replacement of H by OH at C-12 was verified by the HMBC correlations of *δ*_H_ 6.82 (H-10) and 6.74 (H-11) with *δ*_C_ 143.3 (C-12). The relative configurations at the various stereogenic centers were established from ROEs and by comparison of the ^13^C NMR spectra with the reported values. The ROESY spectrum (Fig. 4) displayed cross peaks of H-3/H-9, H-3/H-5b, H-3/H-6b, H-3/H_2_-14, H_2_-14/H-15 indicating the relative configurations of C-7 and C-3, which also in turn allowed the orientation of H-15 to be assigned as *α*. The preferred boat conformation adopted by the piperidine ring D could be deduced from the observed H-14a/H-21a ROE correlation [16]. Likewise, the key ROE correlations of H-9/H-6b and H-6a/H-20 suggested a *β* orientation for H-20. Characteristic chemical shifts of C-16 (*δ* 105.6) and C-19 (69.0) suggested that the configuration of C-19 in **4** should be *R*,which was consistent with that of alstogustine (*δ*_C_ 105.3, 68.8) [17], rather than that of 19-*epi*-alstogustine (*δ*_C_ 104.8, 70.6) [17]. On the basis of the above evidences, the structure of **4** was deduced to be as shown in Fig. 1 and named as alstoniascholarine O.

**Table 3:** ^1^H and ^13^C NMR spectroscopic data for alkaloids **4–6** in CD_3_OD^a^

Position	**4**		**5**		**6**	
	*δ*_C_	*δ*_H_ (*J* inHz)	*δ*_C_	*δ*_H_ (*J* in Hz)	*δ*_C_	*δ*_H_ (*J* inHz)
2	165.6		164.8		166.2	
3	61.8	4.58 br s	78.0	4.83 br s	78.4	4.33 br s
5a	53.0	3.70 m	66.8	4.08 2H m	69.1	3.75 m
5b		3.43 m				3.68 m
6a	43.0	2.56 m	41.0	2.59 m	41.0	2.50 m
6b		2.19 m		2.38 m		2.24 m
7	57.9		57.1		57.0	
8	134.5		134.1		135.2	
9	113.0	6.89 d (7.5)	113.0	7.00 d (7.4)	114.0	7.10 d (7.4)
10	123.7	6.82 t (7.8)	123.8	6.85 t (7.8)	123.8	6.97 t (8.0)
11	117.4	6.74 d (8.1)	117.6	6.76 d (8.1)	112.5	6.92 d (8.2)
12	143.3		143.4		146.2	
13	132.7		132.8		133.6	
14a	26.5	2.37 dt (14.6, 3.4)	25.9	2.62 m	25.8	2.65 br d (14.5)
14b		1.35 dt (14.6, 2.5)		1.47 br d (14.8)		1.29 br d (14.5)
15	25.8	3.32 m	25.1	3.43 br s	25.7	3.36 br s
16	105.3		105.0		105.6	
18	20.5	1.32 d (6.6)	20.8	1.37 d (6.4)	20.8	1.33 d (6.4)
19	69.0	3.82 m	68.4	3.91 m	69.0	3.95 m
20	42.0	2.10 m	44.4	2.13 m	44.8	1.97 m
21a	48.5	3.48 dd (13.5, 11.7)	64.1	4.00 dd (13.4, 6.1)	66.3	3.71 dd (13.6, 6.1)
21b		3.22 dd (13.5, 6.2)		3.81 dd (13.0, 10.6)		3.47 dd (13.4, 9.3)
12-OMe					56.3	3.88 s
CO_2_Me	168.4		168.1		168.4	
CO_2_ Me	51.7	3.79, s	51.8	3.79 s	51.8	3.78 s

^a^Recorded at 600 and 150 MHz

Alkaloid **5** exhibited a molecular ion peak at *m*/*z* 372.1676 in its HREIMS spectrum, indicating the molecular formula of C_20_H_24_N_2_O_5_ with sixteen mass units higher than that of **4**. Examination of the ^1^H NMR spectrum revealed four downfield shifts for the protons H-3 (*δ*_H_ 4.83), H-5 (*δ*_H_ 4.08), and H-21 (*δ*_H_ 4.00 and 3.81), while the ^13^C NMR data exhibited noticeable downfield shifts involving C-3 (*δ*_C_ 78.0), C-5 (*δ*_C_ 66.8), and C-21 (*δ*_C_ 64.1), when compared to those of **4**. These features are characteristic of *N*(4)-oxides, and this conclusion was further confirmed by the HMBC correlations of *δ*_H_ 4.08 (H_2_-5), 2.62 (H-14a), and 4.00 (H-21a) with *δ*_C_ 78.0 (d, C-3). The ROESY correlations (Fig. 4) indicated that the relative configuration of **5** was the same as that of **4**. Other parts of **5** were identical to those of **4** as secured by detailed analysis of extensive 2D NMR spectral data of **5**. Thus, the structure of alkaloid **5** was determined to be as shown in Fig. 1 and named alstoniascholarine P.

Alkaloid **6** was deduced to have the molecular formula C_21_H_26_N_2_O_5_, as indicated by the observed ion peak at *m*/*z* 386.1847 [M]^+^ in its HREIMS data. The IR, UV and 1D NMR spectra of **6** were similar to those of **5**, which suggested that **6** was also a strychnan type indole alkaloid. The ^1^H and ^13^C NMR data (Table 3) of **6** exhibited high similarities with those of *N*^b^-demethylalstogustine *N*-oxide (**25**), except for the loss of one aromatic proton and the existence of an additional methoxy group (*δ*_C_ 56.3, *δ*_H_ 3.88). By comparing the ^1^H and ^13^C NMR spectral data of **6** with those of **25**, the C-12 carbon signal was significantly deshielded, while the C-11 and C-13 carbon signals were relatively shielded, suggesting that the methoxy was attached to C-12. This conclusion was further confirmed by the correlation of methoxy protons at *δ*_H_ 3.88 with C-12 (*δ*_C_ 146.2) in the HMBC spectrum. Complete analysis of 2D NMR spectral data of **6** suggested that its other parts were the same to those of **4** and **5**. Hence, the structure of alkaloid **6** was assigned as shown in Fig. 1 and named as alstoniascholarine Q.

The known alkaloids picrinine (**7**) [18], strictamine (**8**) [19], tubotawine (**9**) [20], alstolucine D (**10**) [8], nareline (**11**) [21], picralinal (**12**) [18], isoalschomine (**13**) [18], polyneuridine (**14**) [22], burnamine (**15**) [23], echitamidine (**16**) [24], scholarisine I (**17**) [25], 19,20-*Z*-vallesamine (**18**) [26], 19,20-*E*-vallesamine (**19**) [26], scholaricine (**20**) [27], 19-*epi*-scholaricine (**21**) [16], pseudoakuammigine *N*^b^-oxide (**22**) [28], tubotawine *N*^b^-oxide (**23**) [29], vallesamine *N*^b^-oxide (**24**) [16], and *N*^b^-demethylalstogustine *N*-oxide (**25**) [30], were identified by comparison of their spectroscopic data with those reported in the literature.

Considering the various bioactivities of the chemical constituents from *A. scholaris* reported previously [5], six new alkaloids (**1**–**6**) were evaluated for their cytotoxic and neurite outgrowth-promoting activities. Unfortunately, the alkaloids **1**–**6** neither showed cytotoxic activity against five human cancer cell lines (SW-480, SMMC-7721, HL-60, MCF-7, and A-549), nor exhibited neurite outgrowth-promoting activity.

## Experimental Section

### General Experimental Procedures

Optical rotations were recorded on a JASCO P-1020 polarimeter. UV spectra were measured on a Shimadzu UV-2401PC spectrophotometer. IR spectra were performed on a Bruker FT-IR Tensor-27 infrared spectrophotometer with KBr discs. NMR spectroscopic data were obtained on Bruker AM-400 and DRX-500 spectrometers using TMS as an internal standard. Unless otherwise specified, chemical shifts (*δ*) were expressed in ppm with reference to the solvent signals. ESIMS spectra were performed on Waters Xevo TQ-S and Bruker HCT/Esquire mass spectrometers; HREIMS and HRESIMS analyses were carried out on Waters AutoSpec Premier P776 and Agilent 6540 UHD Accurate-Mass Q-TOF LC/MS mass spectrometers, respectively. Column chromatography (CC) was conducted on silica gel (200–300 mesh, Qingdao Marine Chemical Co., Ltd., Qingdao, People’s Republic of China), RP-18 gel (20–45 μm, Fuji Silysia Chemical Ltd., Japan), and Sephadex LH-20 (GE Healthcare Bio-sciences AB, Sala, Sweden). Fractions were monitored by TLC (GF 254, Qingdao Marine Chemical Co., Ltd., Qingdao), and spots were visualized by Dragendorff’s reagent.

### Plant Material

The inadequately dried leaves of *A. scholaris* were collected in June 2013 in Pu’er city of Yunnan Province, P. R. China and identified by Dr. Xiao-Dong Luo, Kunming Institute of Botany, Chinese Academy of Sciences. A voucher specimen (No. Luo20130720) has been deposited in the State Key Laboratory of Phytochemistry and Plant Resources in West China, Kunming Institute of Botany, Chinese Academy of Sciences.

### Extraction and Isolation

The inadequately dried leaves (40 kg) of *A. scholaris* were extracted with MeOH at room temperature, and the solvent was evaporated *in vacuo*. The extract was dissolved in 0.3 % HCl solution and then partitioned with EtOAc three times. The acidic water-soluble material, subsequently basified with 5 % ammonia solution to pH 9–10, was repeatedly partitioned with EtOAc three times to give the crude alkaloid extract (155 g). The alkaloid extract was subjected to a silica gel column and eluted with CHCl_3_–MeOH (30:1, 15:1, 10:1, 5:1, 1:1) to afford fractions I–V. Fraction I (40.0 g) was separated by RP-18 CC, eluted with MeOH-H_2_O (55:1 → 100:1) to give five subfractions (Fr. I–I to Fr. I–V). Fraction I–II (3.8 g) was further separated by silica gel CC, eluted with CHCl_3_-acetone (10:1 → 5:1) to yield alkaloids **8** (25 mg), **9** (18 mg), **10** (38 mg), and **11** (123 mg). Fraction I–III (16 g) was subjected to RP-18 CC, eluted with MeOH-H_2_O (75:25 → 95:5) to obtain alkaloids **1** (18 mg), **2** (20 mg), and **7** (9.8 g). Fraction II (10 g) was separated by RP-18 CC, eluted with MeOH-H_2_O (50:50–80:20) and subsequently purified by Sephadex LH-20 CC to yield alkaloids **12** (17 mg), **13** (12 mg), **14** (10 mg), **15** (15 mg) and **16** (32 mg). Fraction III (35 g) was subjected to RP-18 CC and eluted with MeOH-H_2_O (40:60 → 65:35) to obtain five fractions (Fr. III–I to Fr. III–V). Fraction III–II (200 mg) was chromatographed repeatedly over silica gel CC, eluted with CHCl_3_–MeOH (12:1 → 10:1) to give alkaloids **3** (12 mg) and **17** (8 mg). In the same way, alkaloid **20** (6.5 g) was obtained from fraction III–III (8.2 g) by silica gel CC and eluted with CHCl_3_–MeOH (12:1 → 10:1). Fraction III–IV (1 g) was separated by RP-18 CC, eluted with MeOH-H_2_O (35:65 → 60:40) to afford alkaloids **4** (7 mg), **18** (45 mg), **19** (18 mg), and **21** (85 mg). Fraction IV (14 g) was separated over RP-18 CC, eluted with MeOH-H_2_O (25:75 → 60:40) to afford five fractions (Fr. IV–I to Fr. IV–V). Fraction IV–II (120 mg) was subjected to silica gel CC, eluted with CHCl_3_–MeOH (8:1 → 5:1) to yield alkaloid **22** (15 mg). Fraction IV–III (200 mg) was purified by RP-18 CC, eluted with MeOH-H_2_O (30:70 → 50:50) to give alkaloids **23** (4 mg) and **24** (6 mg). Fraction IV–IV (300 mg) was subjected to RP-18 CC eluted with MeOH-H_2_O (25:75 → 50:50) and further purified by silica gel CC eluted with CHCl_3_–MeOH (6:1 → 4:1) to yield alkaloids **5** (6 mg), **6** (8 mg) and **25** (6 mg).

#### Alstoniascholarine L (**1**)

White amorphous powder; $\left[ \alpha \right]_{D}^{24}$ –121.1 (*c* 0.1 MeOH); UV (MeOH) *λ*_max_ (log *ε*) 204 (4.45), 240 (3.87), 298 (3.49) nm; IR (KBr) *ν*_max_ 3440, 2924, 1748, 1630, 1612, 1473, 1090 cm^−1^; CD (MeOH) *λ*_max_ (Δ*ε*) 202 (+8.1), 239 (–10.8), 302 (–2.1) nm; ^1^H (400 MHz, CD_3_OD) and ^13^C NMR (100 MHz, CD_3_OD) data, see Table 2; ESIMS *m*/*z* 379 [M + Na]^+^; HREIMS *m*/*z* 356.1366 ([M]^+^ calcd for C_19_H_20_N_2_O_5_, 356.1372).

#### Alstoniascholarine M (**2**)

White amorphous powder; $\left[ \alpha \right]_{D}^{24}$ –117.1 (*c* 0.1 MeOH); UV (MeOH) *λ*_max_ (log *ε*) 204 (4.48), 240 (3.90), 298 (3.52) nm; IR (KBr) *ν*_max_ 3441, 2923, 1748, 1631, 1474, 1241, 1085 cm^−1^; CD (MeOH) *λ*_max_ (Δ*ε*) 202 (+8.3), 239 (–11.6), 302 (–2.4) nm; ^1^H (400 MHz, CD_3_OD) and ^13^C NMR (100 MHz, CD_3_OD) data, see Table 2; ESIMS *m*/*z* 379 [M + Na]^+^; HREIMS *m*/*z* 356.1358 ([M]^+^ calcd for C_19_H_20_N_2_O_5_, 356.1372).

#### Alstoniascholarine N (**3**)

White amorphous powder; $\left[ \alpha \right]_{D}^{23}$ +2.7 (*c* 0.1 MeOH); UV (MeOH) *λ*_max_ (log *ε*) 204 (4.38), 229 (4.35), 291 (3.63) nm; IR (KBr) *ν*_max_ 3426, 2976, 1764, 1640, 1511, 1244, 1104 cm^−1^; ^1^H (600 MHz, CD_3_OD) and ^13^C NMR (150 MHz, CD_3_OD) data, see Table 2; ESIMS *m*/*z* 377 [M + Na]^+^; HREIMS *m*/*z* 354.1577 ([M]^+^ calcd for C_20_H_22_N_2_O_4_, 354.1580).

#### Alstoniascholarine O (**4**)

White amorphous powder; $\left[ \alpha \right]_{D}^{23}$ –348.7 (*c* 0.1 MeOH); UV (MeOH) *λ*_max_ (log *ε*) 213 (4.09), 230 (4.00), 289 (3.61), 337 (4.06) nm; IR (KBr) *ν*_max_ 3417, 2977, 1678, 1620, 1440, 1260, 1074 cm^−1^; ^1^H (600 MHz, CD_3_OD) and ^13^C NMR (150 MHz, CD_3_OD) data, see Table 3; ESIMS *m*/*z* 357 [M + H]^+^; HRESIMS *m*/*z* 357.1810 ([M + H]^+^ calcd for C_20_H_25_N_2_O_4_, 357.1809).

#### Alstoniascholarine P (**5**)

White amorphous powder; $\left[ \alpha \right]_{D}^{23}$ –329.3 (*c* 0.1 MeOH); UV (MeOH) *λ*_max_ (log *ε*) 211 (4.12), 287 (3.60), 336 (3.98) nm; IR (KBr) ν_max_ 3431, 2977, 1680, 1621, 1439, 1202, 1059 cm^−1^; ^1^H (600 MHz, CD_3_OD) and ^13^C NMR (150 MHz, CD_3_OD) data, see Table 3; ESIMS *m*/*z* 373 [M + H]^+^; HREIMS *m*/*z* 372.1676 ([M]^+^ calcd for C_20_H_24_N_2_O_5_, 372.1685).

#### Alstoniascholarine Q (**6**)

White amorphous powder; $\left[ \alpha \right]_{D}^{24}$ –298.7 (*c* 0.12 MeOH); UV (MeOH) *λ*_max_ (log *ε*) 210 (4.21), 287 (3.76), 334 (4.06) nm; IR (KBr) ν_max_ 3438, 2965, 1681, 1615, 1461, 1266, 1060 cm^−1^; ^1^H (600 MHz, CD_3_OD) and ^13^C NMR (150 MHz, CD_3_OD) data, see Table 3; ESIMS *m*/*z* 387 [M + H]^+^; HREIMS *m*/*z* 386.1847 ([M]^+^ calcd for C_21_H_26_N_2_O_5_, 386.1842).

### Antitussive Effects Against Ammonia-Induced Cough

The antitussive assay was performed using the technique as described in the literature [31]. ICR (Institute of Cancer Research) mice of either sex (19–24 g) were purchased from Kunming Medical College (license number SYXK 2005-0001). All mice were housed at room temperature (20–25 °C) and constant humidity (40–70 %) under a 12 h light–dark cycle in SPF (Specific Pathogen Free) grade laboratory. The animal study was performed according to the international rules considering animal experiments and the internationally accepted ethical principles for laboratory animal use and care. The mice were divided randomly, of which 12 mice per group. The negative control group of animals was treated with distilled water orally, and the positive control was treated with codeine phosphate, the remaining groups were treated with test samples. Antitussive activity was investigated on a classical mouse cough model induced by ammonia liquor. Briefly, each mouse was placed in a 300 mL special glass chamber and exposed to 40 μL 25 % ammonia solution. The cough frequency produced during 2 min exposure period was counted. In the second assay for alkaloids, cough frequency and latent period of cough were recorded.

### Cytotoxic Activity Assay

The following human cancer cell lines were used: SW-480, SMMC-7721, HL-60, MCF-7, and A-549. All cells were cultured in RPMI-1640 or DMEM medium (Hyclone, Logan, UT), supplemented with 10 % fetal bovine serum (Hyclone) at 37 °C in a humidified atmosphere with 5 % CO_2_. Cell viability was assessed by conducting colorimetric measurements of the amount of insoluble formazan formed in living cells based on the reduction of 3-(4, 5-dimethylthiazol-2-yl)-2, 5-diphenyltetrazolium bromide (MTT) (Sigma, St. Louis, MO) [32]. Briefly, 100 μL of adherent cells was seeded into each well of a 96-well cell culture plate and allowed to adhere for 12 h before drug addition, while suspended cells were seeded just before drug addition, both with an initial density of 1 × 10^5^ cells/mL in 100 μL of medium. Each cell line was exposed to the test compound at various concentrations in triplicate for 48 h, with cisplatin and paclitaxel (Sigma) as positive controls. After the incubation, MTT (100 μg) was added to each well, and the incubation continued for 4 h at 37 °C. The cells were lysed with 100 μL of 20 % SDS-50 % DMF after removal of 100 μL of medium. The optical density of the lysate was measured at 595 nm in a 96-well Microtiter plate reader (Bio-Rad 680).

### Neurite Outgrowth-Promoting Activity Assay

The neurotrophic activities of the test compounds were examined according to an assay using PC12 cells as reported elsewhere [33]. Briefly, PC12 cells were maintained in F12 medium supplemented with 12.5 % horse serum (HS), and 2.5 % fetal bovine serum (FBS), and incubated at 5 % CO_2_ and 37 °C. Test compounds were dissolved in DMSO. For the neurite outgrowth-promoting activity bioassay, PC12 cells were seeded at a density of 5 × 10^4^ cells/mL in 48-well plate coated with poly-l-lysine. After 24 h, the medium was changed to that containing 10 µM of each test compounds plus 5 ng/mL NGF, or various concentrations of NGF (50 ng/mL for the positive control, 5 ng/mL for the negative control). The final concentration of DMSO was 0.05 %, and the same concentration of DMSO was added into the negative control. After 72 h incubation, the neurite outgrowth was assessed under a phase contrast microscope. Neurite processes with a length equal to or greater than the diameter of the neuron cell body were scored as neurite bearing cells. The ratio of the neurite-bearing cells to total cells (with at least 100 cells examined/view area; 5 viewing area/well) was determined and expressed as a percentage.

## Electronic supplementary material

Supplementary material 1 (PDF 3684 kb)

## References

[1] Atta-ur-Rahman, Alvi KA (1987). Phytochemistry.

[2] Kam TS, Nyeoh KT, Sim KM, Yoganathan K (1997). Phytochemistry.

[3] Macabeo APG, Krohn K, Gehle D, Read RW, Brophy JJ, Cordell GA, Franzblau SG, Aguinaldo AM (2005). Phytochemistry.

[4] Zhang L, Zhang CJ, Zhang DB, Wen J, Zhao XW, Li Y, Gao K (2014). Tetrahedron Lett..

[5] Khyade MS, Kasote DM, Vaikos NP (2014). J. Ethnopharmacol..

[6] Jagetia GC, Baliga MS (2005). J. Ethnopharmacol..

[7] Khan MR, Omoloso AD, Kihara M (2003). Fitoterapia.

[8] Shang JH, Cai XH, Feng T, Zhao YL, Wang JK, Zhang LY, Yan M, Luo XD (2010). J. Ethnopharmacol..

[9] Channa S, Dar A, Ahmed S, Atta-ur-Rahman (2005). J. Ethnopharmacol..

[10] Cai XH, Du ZZ, Luo XD (2007). Org. Lett..

[11] Cai XH, Tan QG, Liu YP, Feng T, Du ZZ, Li WQ, Luo XD (2008). Org. Lett..

[12] Cai XH, Liu YP, Feng T, Luo XD (2008). Chin. J. Nat. Med..

[13] Feng T, Cai XH, Zhao PJ, Du ZZ, Li WQ, Luo XD (2009). Planta Med..

[14] Yang XW, Qin XJ, Zhao YL, Lunga PK, Li XN, Jiang SZ, Cheng GG, Liu YP, Luo XD (2014). Tetrahedron Lett..

[15] Qin XJ, Zhao YL, Lunga PK, Yang XW, Song CW, Cheng GG, Liu L, Chen YY, Liu YP, Luo XD (2015). Tetrahedron.

[16] Yamauchi T, Abe F, Chen RF, Nonaka GI, Santisuk T, Padolina WG (1990). Phytochemistry.

[17] Kuehne ME, Xu F, Brook CS (1994). J. Org. Chem..

[18] Abe F, Chen RF, Yamauchi T, Marubayashi N, Ueda I (1989). Chem. Pharm. Bull..

[19] Atta-ur-Rahman Habib-ur-Rehman (1986). Planta Med..

[20] Yamauchi T, Abe F, Padolina WG, Dayrit FM (1990). Phytochemistry.

[21] Morita Y, Hesse M, Schmid H, Banerji A, Banerji J, Chatterjee A, Oberhänsli WE (1977). Helv. Chim. Acta.

[22] Antonaccio LD, Pereira NA, Gilbert B, Vorbrueggen H, Budzikiewicz H, Wilson JM, Durham LJ, Djerassi C (1962). J. Am. Chem. Soc..

[23] Burnell RH, Medina JD (1968). Phytochemistry.

[24] Keawpradub D, Takayama H, Aimi N, Sakai SI (1994). Phytochemistry.

[25] Yang XW, Luo XD, Lunga PK, Zhao YL, Qin XJ, Chen YY, Liu L, Li XN, Liu YP (2015). Tetrahedron.

[26] Atta-ur-Rahman KA, Alvi SA, Abbas W (1987). Voeter. Heterocycles.

[27] Atta-ur-Rahman, Asif M, Ghazala M, Fatima J, Alvi KA (1985). Phytochemistry.

[28] Hu WL, Zhu JP, Hesse M (1989). Planta Med..

[29] Pinar M, Renner U, Hesse M, Schmid H (1972). Helv. Chim. Acta.

[30] Salim AA, Garson MJ, Craik DJ (2004). J. Nat. Prod..

[31] Xu SY, Bian RL, Chen X (1991). Pharmacological Experiment Methodology.

[32] Mosmann T (1983). J. Immunol. Methods.

[33] Pradines A, Magazin M, Schiltz P, Le Fur G, Caput D, Ferrara P (1995). J. Neurochem..

