# Supplementary material for: Monoterpenoid Indole Alkaloids from Inadequately Dried Leaves of Alstonia scholaris
Source: Nat Prod Bioprospect. 2015 Aug 18;5(4):185–93. doi: 10.1007/s13659-015-0066-2 (PMC4567994; doi:10.1007/s13659-015-0066-2)
Supplement: Supplementary file 1 — Supplementary material 1 (PDF 3684 kb) [file 13659_2015_66_MOESM1_ESM.pdf]

Supporting Information for

Monoterpenoid Indole Alkaloids from Inadequately Dried Leaves of *Alstonia scholaris*

Xu-Jie Qin[1,2], Yun-Li Zhao[1], Chang-Wei Song[1], Bei Wang[1,2], Ying-Ying Chen[1,2], Lu Liu[1,2], Qiong-Li[1,2], Dan-Li[1,2], Ya-Ping Liu[1*], Xiao-Dong Luo[1*]

Affiliation

[1]State Key Laboratory of Phytochemistry and Plant Resources in West China, Kunming Institute of Botany, Chinese Academy of Sciences, Kunming 650201, P. R. China

[2]University of Chinese Academy of Science, Beijing 100049, P. R. China

Correspondence

Xiao-Dong Luo; Ya-Ping Liu

State Key Laboratory of Phytochemistry and Plant Resources in West China, Kunming Institute of Botany, Chinese Academy of Sciences, 132# Lanhei Road, Kunming 650201, P. R. China.

Phone: +86 871 6522 3177

Fax: +86 871 6515 0227

xdluo@mail.kib.ac.cn; liuyaping@mail.kib.ac.cn

Contents of Supplementary material	Page
**Fig. 1S** HPLC profiles of total alkaloids from *A. scholaris* leaves	2
**Figs. 2S–7S** NMR spectra of Alstoniascholarine L (**1**)	3–5
**Fig. 8S** HREIMS spectrum of Alstoniascholarine L (**1**)	6
**Fig. 9S** CD spectrum of Alstoniascholarine L (**1**)	6
**Figs. 10S–15S** NMR spectra of Alstoniascholarine M (**2**)	7–9
**Fig. 16S** HREIMS spectrum of Alstoniascholarine M (**2**)	10
**Fig. 17S** CD spectrum of Alstoniascholarine M (**2**)	10
**Figs. 18S–23S** NMR spectra of Alstoniascholarine N (**3**)	11–13
**Fig. 24S** HREIMS spectrum of Alstoniascholarine N (**3**)	14
**Figs. 25S–30S** NMR spectra of Alstoniascholarine O (**4**)	14–17
**Fig. 31S** HRESIMS spectrum of Alstoniascholarine O (**4**)	17
**Figs. 32S–37S** NMR spectra of Alstoniascholarine P (**5**)	18–20
**Fig. 38S** HRESIMS spectrum of Alstoniascholarine P (**5**)	21
**Figs. 39S–44S** NMR spectra of Alstoniascholarine Q (**6**)	21–24
**Fig. 45S** HRESIMS spectrum of Alstoniascholarine P (**6**)	24

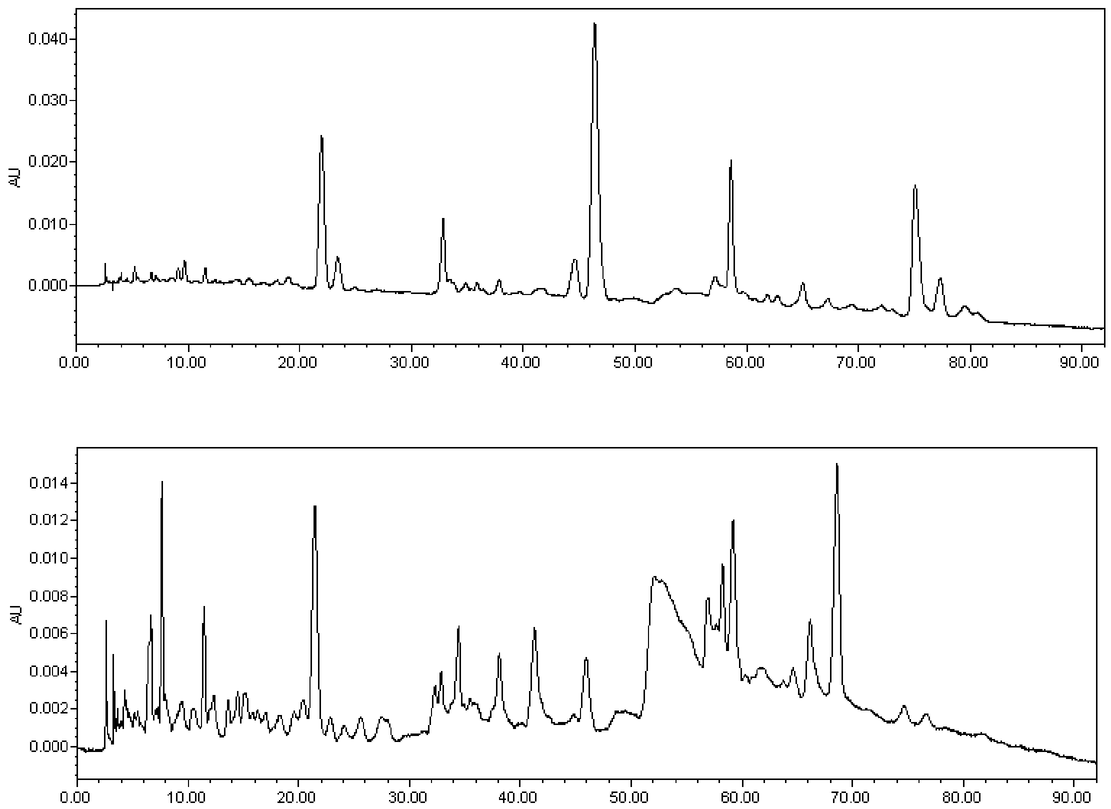

Fig. 1S HPLC profiles of total alkaloids from *A. scholaris* [dried (upper) and inadequately dried (lower) leaves.

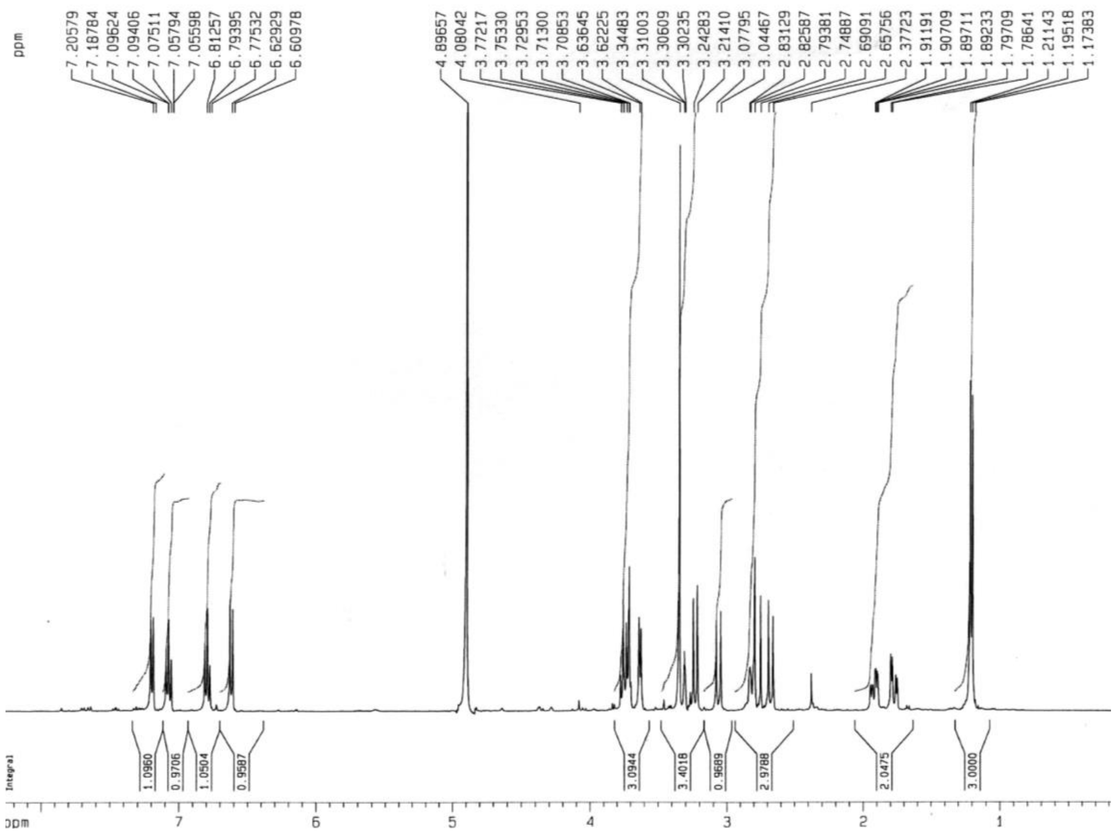

Fig. 2S ^{1}H NMR spectrum of Alstoniascholarine L (**1**) recorded at 400 MHz in CD_3OD

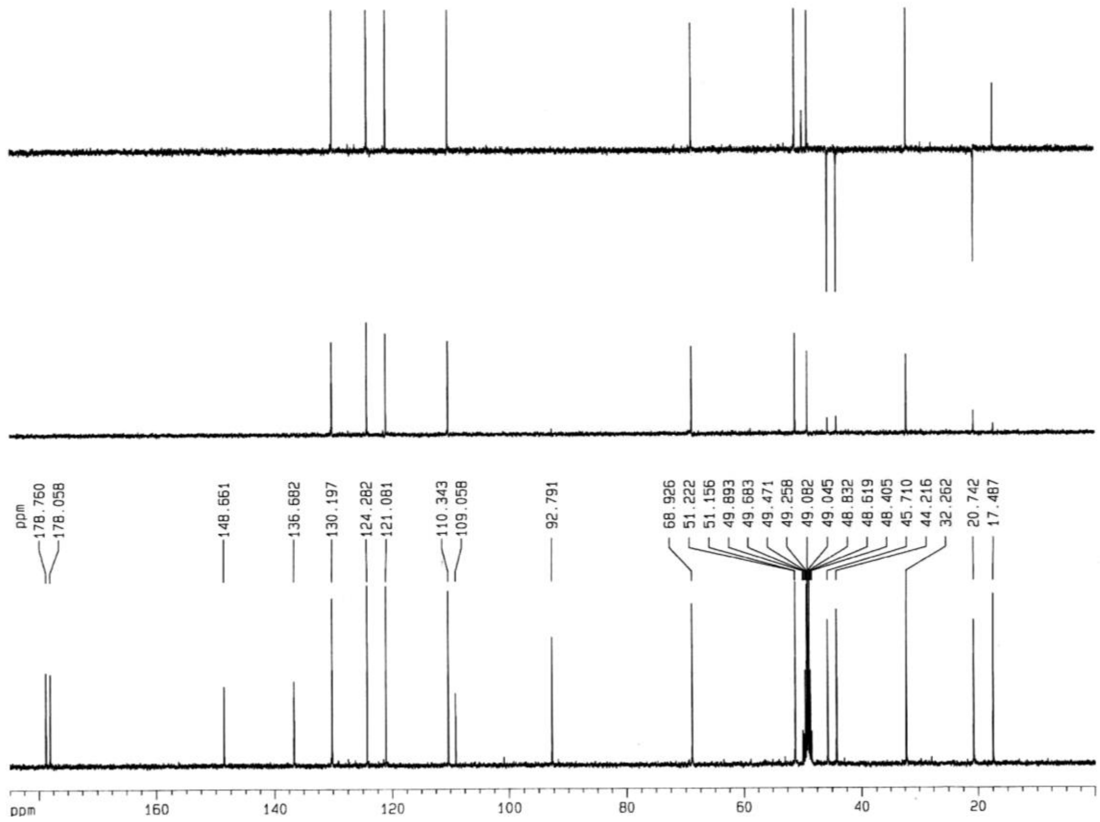

Fig. 3S ^{13}C NMR spectrum of Alstoniascholarine L (**1**) recorded at 100 MHz in CD_3OD

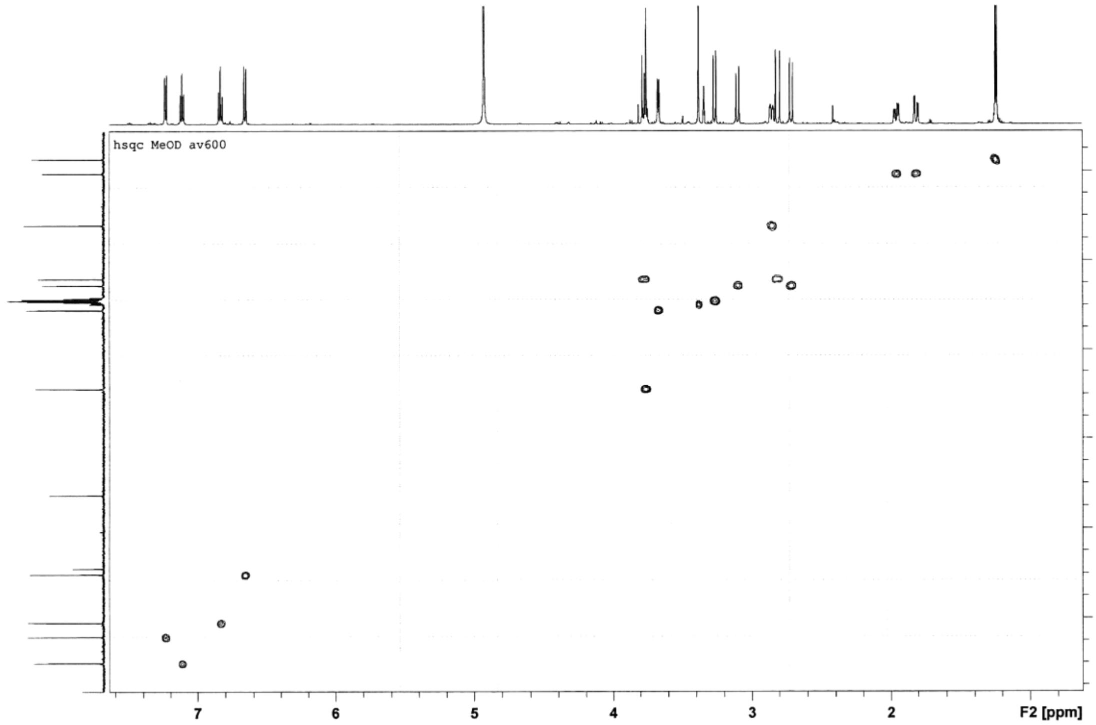

Fig. 4S HSQC spectrum of Alstoniascholarine L (**1**)

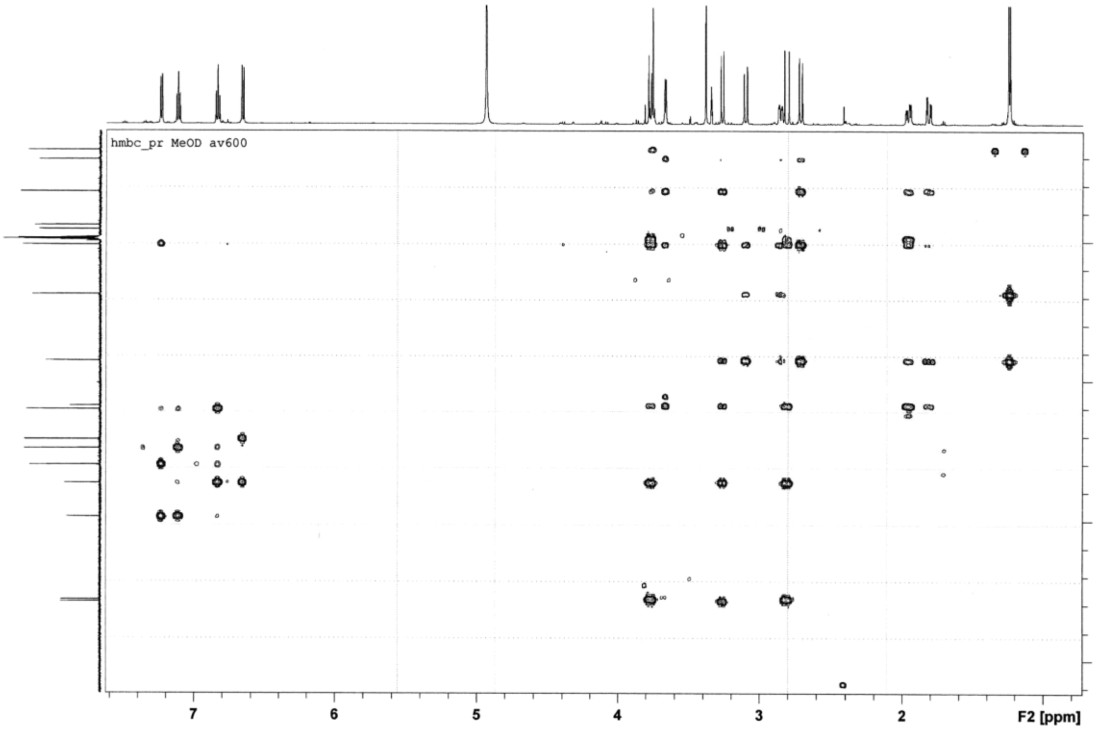

Fig. 5S HMBC spectrum of Alstoniascholarine L (**1**)

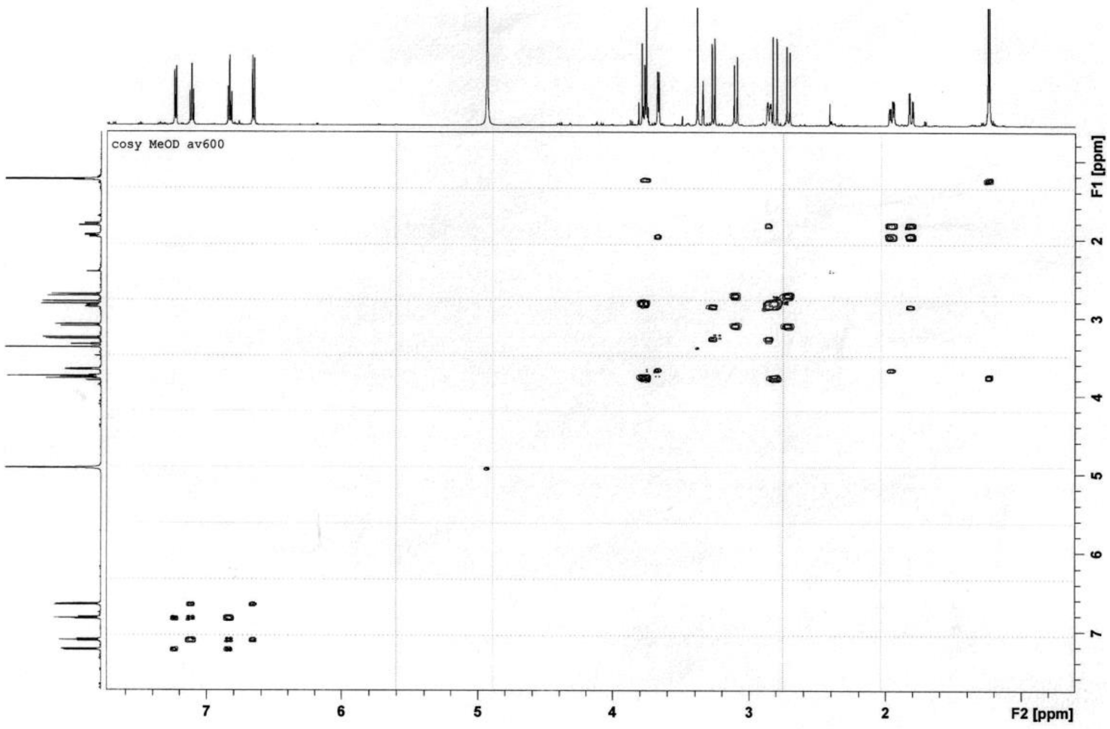

Fig. 6S ^{1}H-^{1}H COSY spectrum of Alstoniascholarine L (**1**)

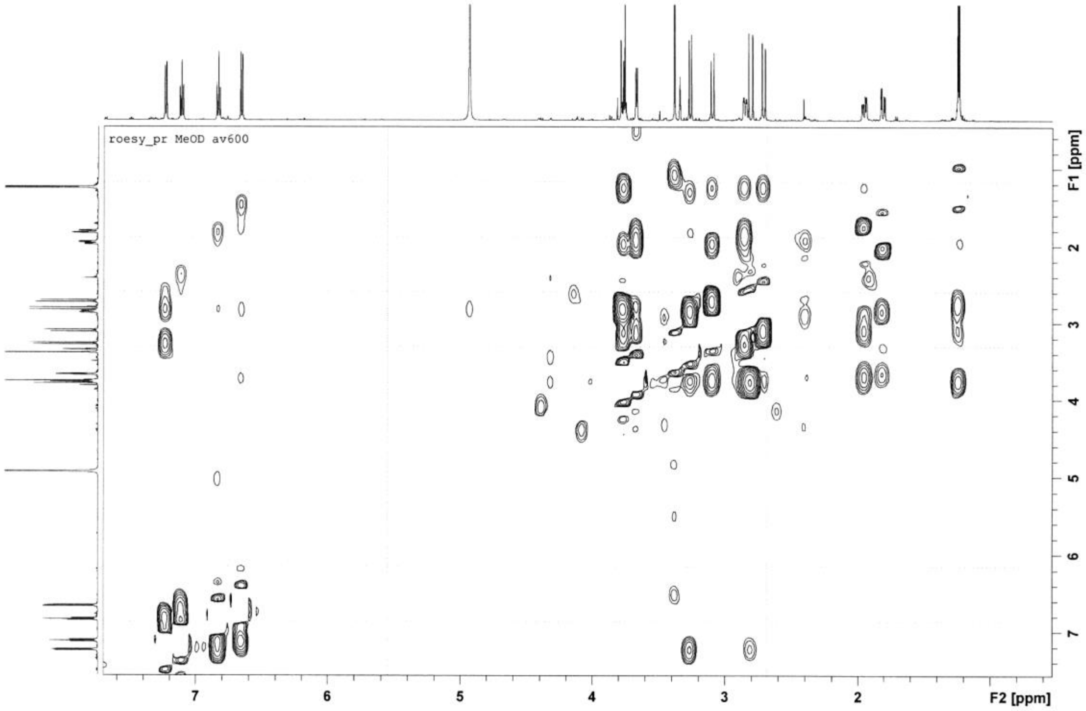

Fig. 7S ROESY spectrum of Alstoniascholarine L (**1**)

Elemental Composition Report **Page 1**

Single Mass Analysis
Tolerance = 10.0 PPM / DBE: min = -10.0, max = 120.0
Selected filters: None

Monoisotopic Mass, Odd and Even Electron Ions
16 formula(e) evaluated with 1 results within limits (up to 51 closest results for each mass)
Elements Used:
C: 0-200 H: 0-400 N: 2-2 O: 4-6

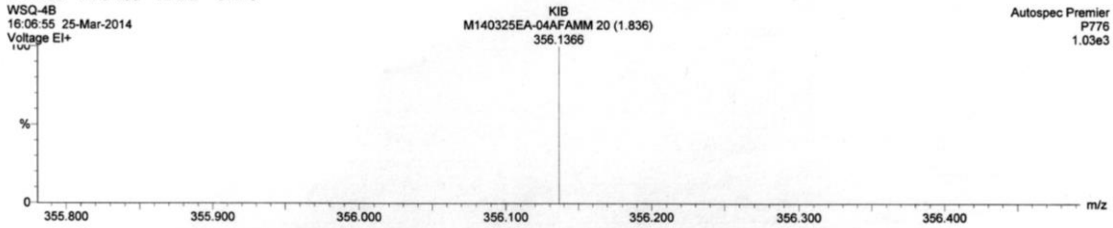

Minimum:				-10.0		
Maximum:		200.0	10.0	120.0		
Mass	Calc. Mass	mDa	PPM	DBE	i-FIT	Formula
356.1366	356.1372	-0.6	-1.7	11.0	5546524.5	C19 H20 N2 O5

Fig. 8S HREIMS spectrum of Alstoniascholarine L (**1**)

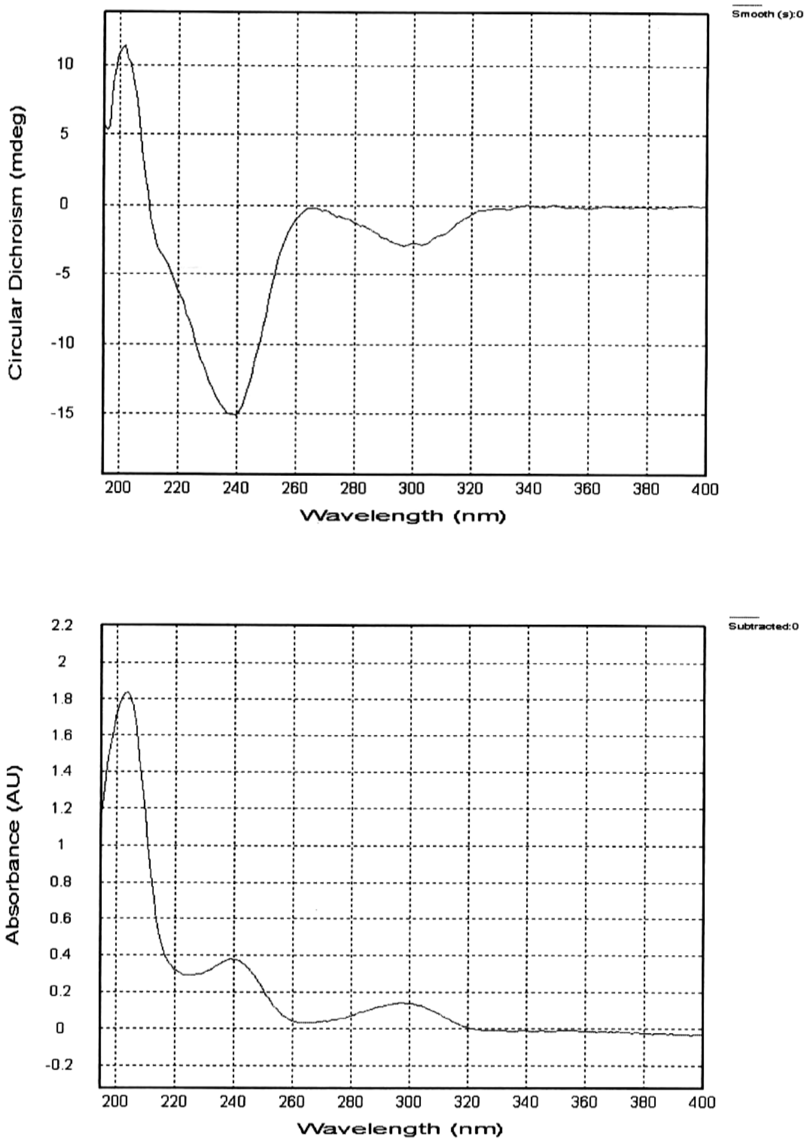

Fig. 9S CD spectrum of Alstoniascholarine L (**1**)

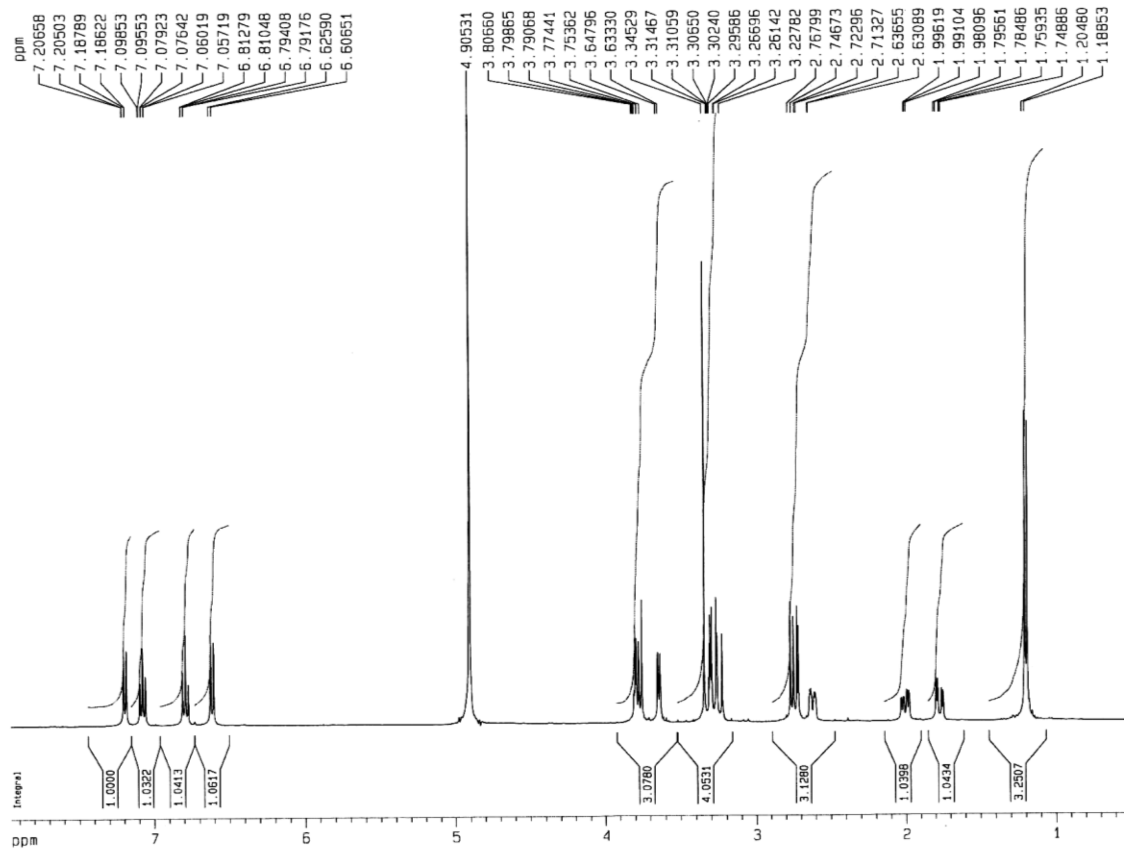

Fig. 10S ^{1}H NMR spectrum of Alstoniascholarine M (**2**) recorded at 400 MHz in CD_3OD

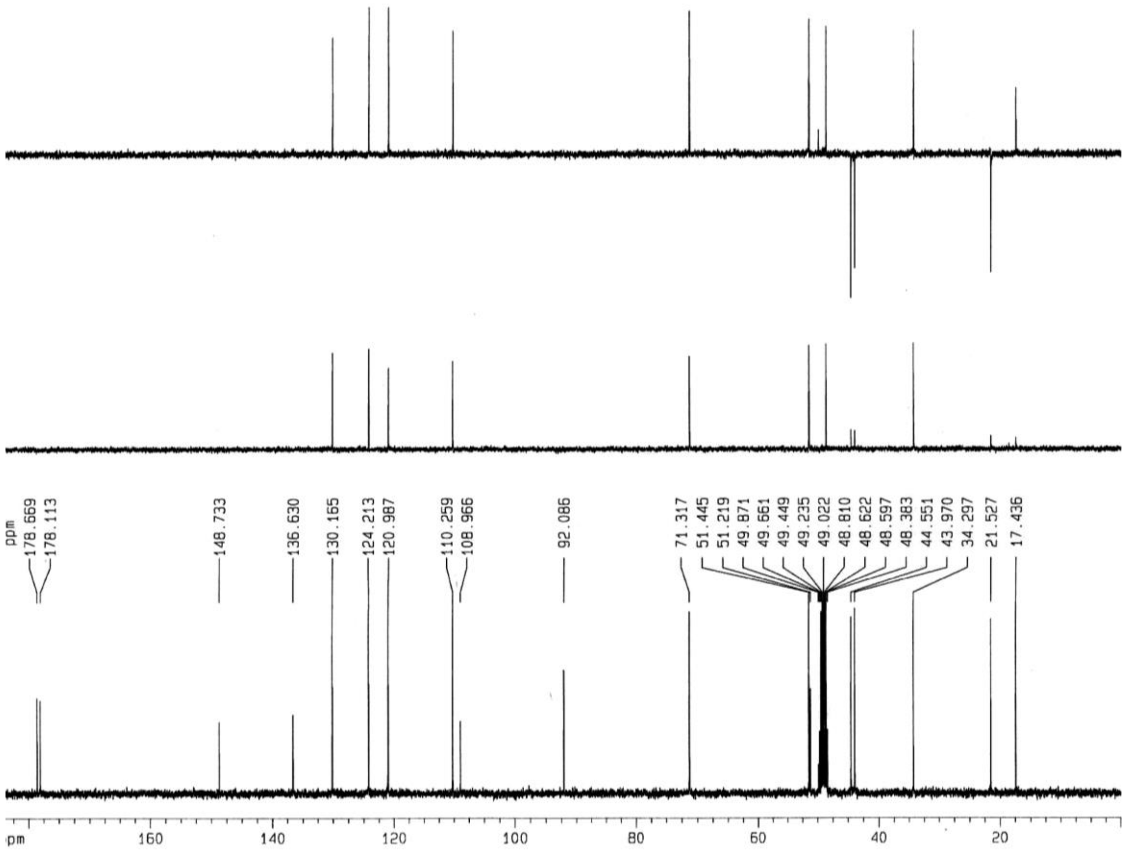

Fig. 11S ^{13}C NMR spectrum of Alstoniascholarine M (**2**) recorded at 100 MHz in CD_3OD

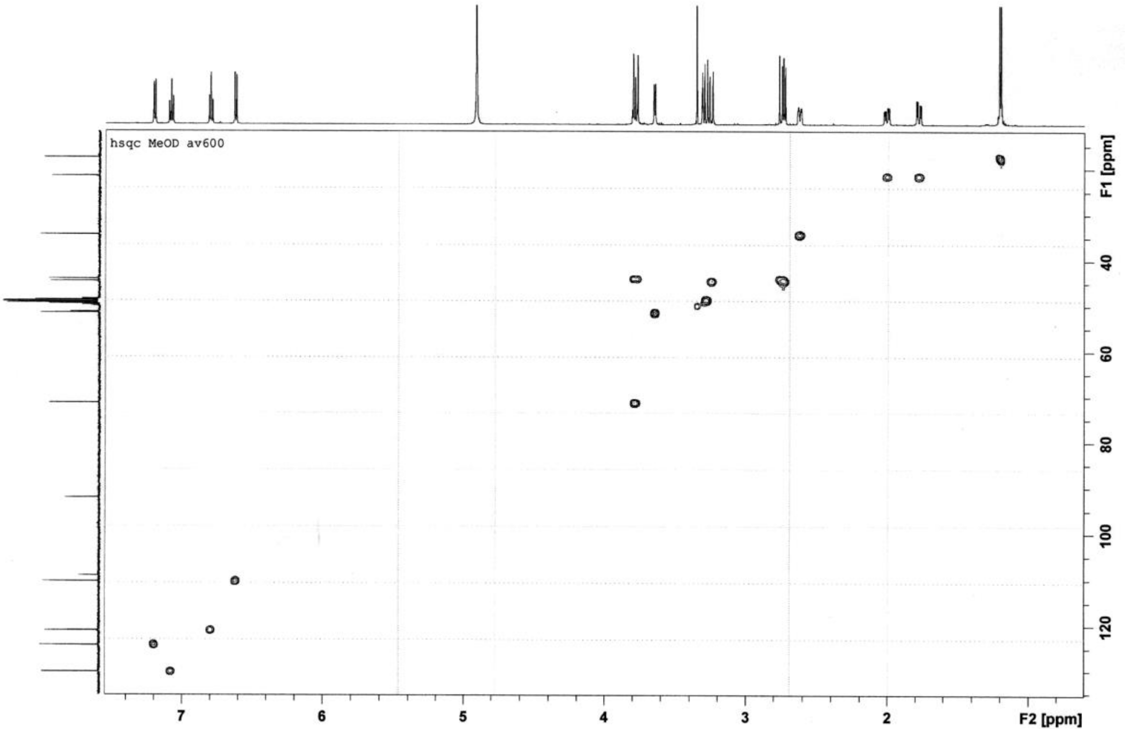

Fig. 12S HSQC spectrum of Alstoniascholarine M (**2**)

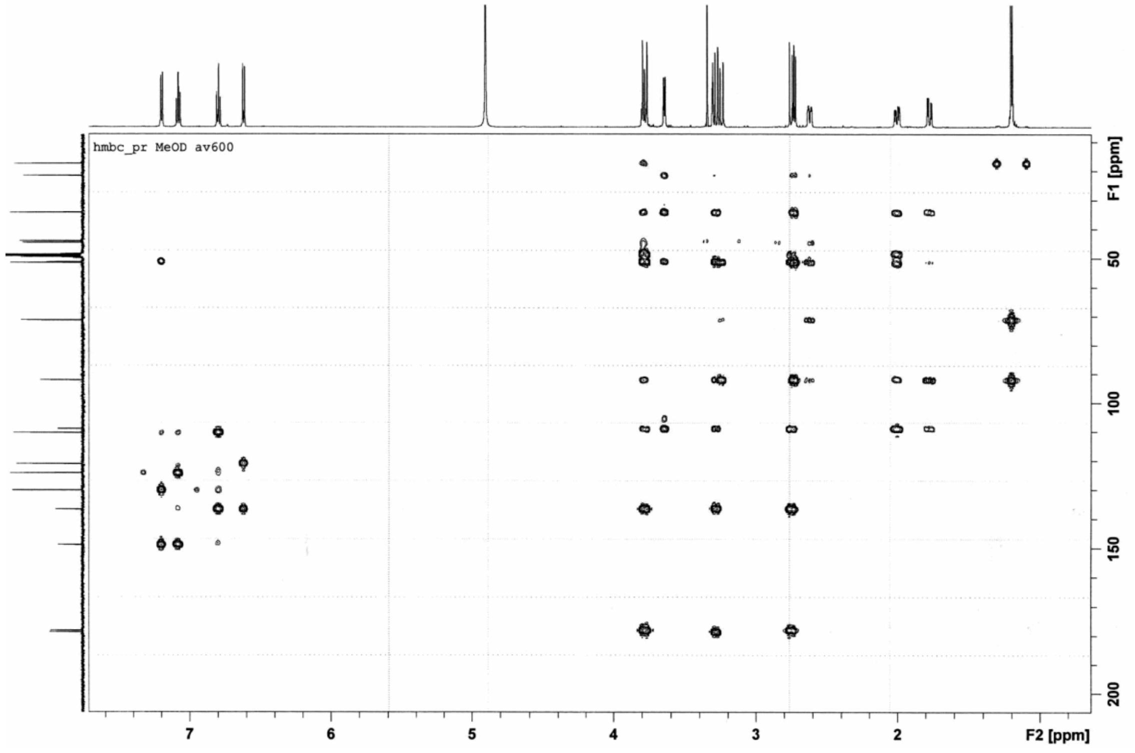

Fig. 13S HMBC spectrum of Alstoniascholarine M (**2**)

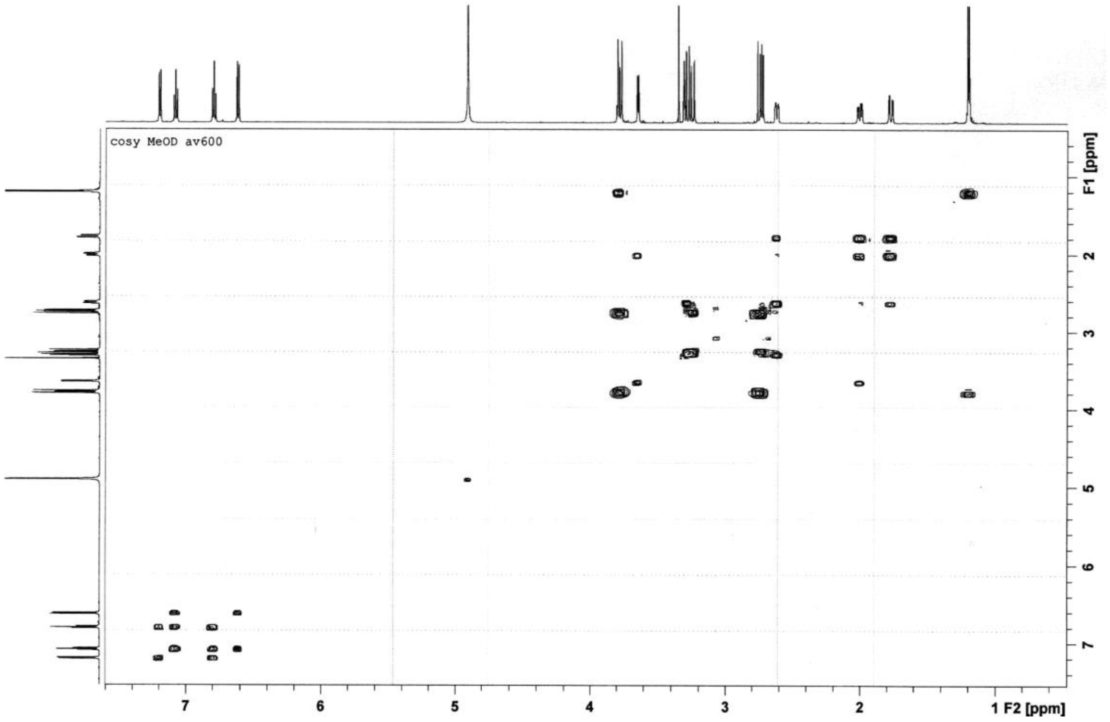

Fig. 14S ^{1}H-^{1}H COSY spectrum of Alstoniascholarine M (**2**)

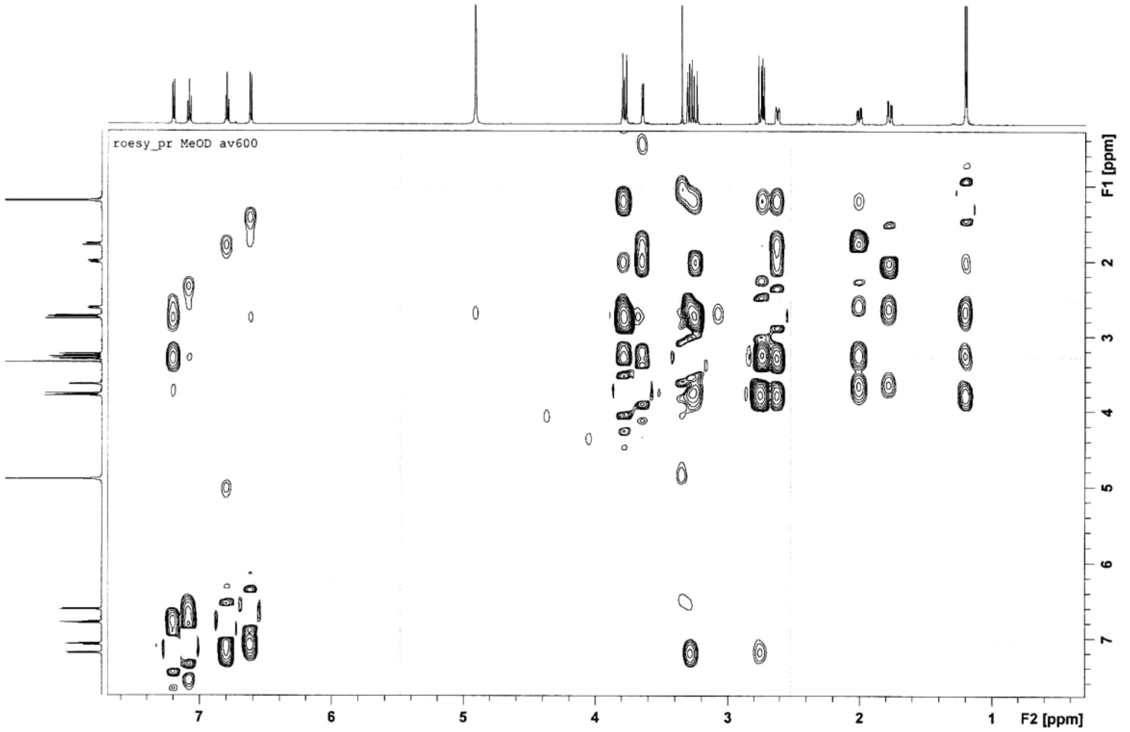

Fig. 15S ROESY spectrum of Alstoniascholarine M (**2**)

Elemental Composition Report **Page 1**

Single Mass Analysis
Tolerance = 10.0 PPM / DBE: min = -10.0, max = 120.0
Selected filters: None

Monoisotopic Mass, Odd and Even Electron Ions
16 formula(e) evaluated with 1 results within limits (up to 51 closest results for each mass)
Elements Used:
C: 0-200 H: 0-400 N: 2-2 O: 4-6

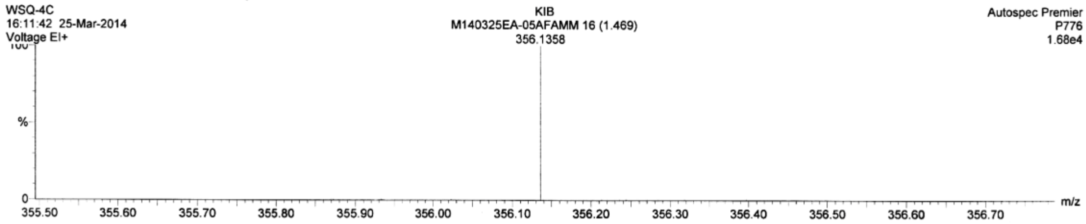

Minimum:				-10.0		
Maximum:		200.0	10.0	120.0		
Mass	Calc. Mass	mDa	PPM	DBE	i-FIT	Formula
356.1358	356.1372	-1.4	-3.9	11.0	5554436.0	C19 H20 N2 O5

Fig. 16S HREIMS spectrum of Alstoniascholarine M (**2**)

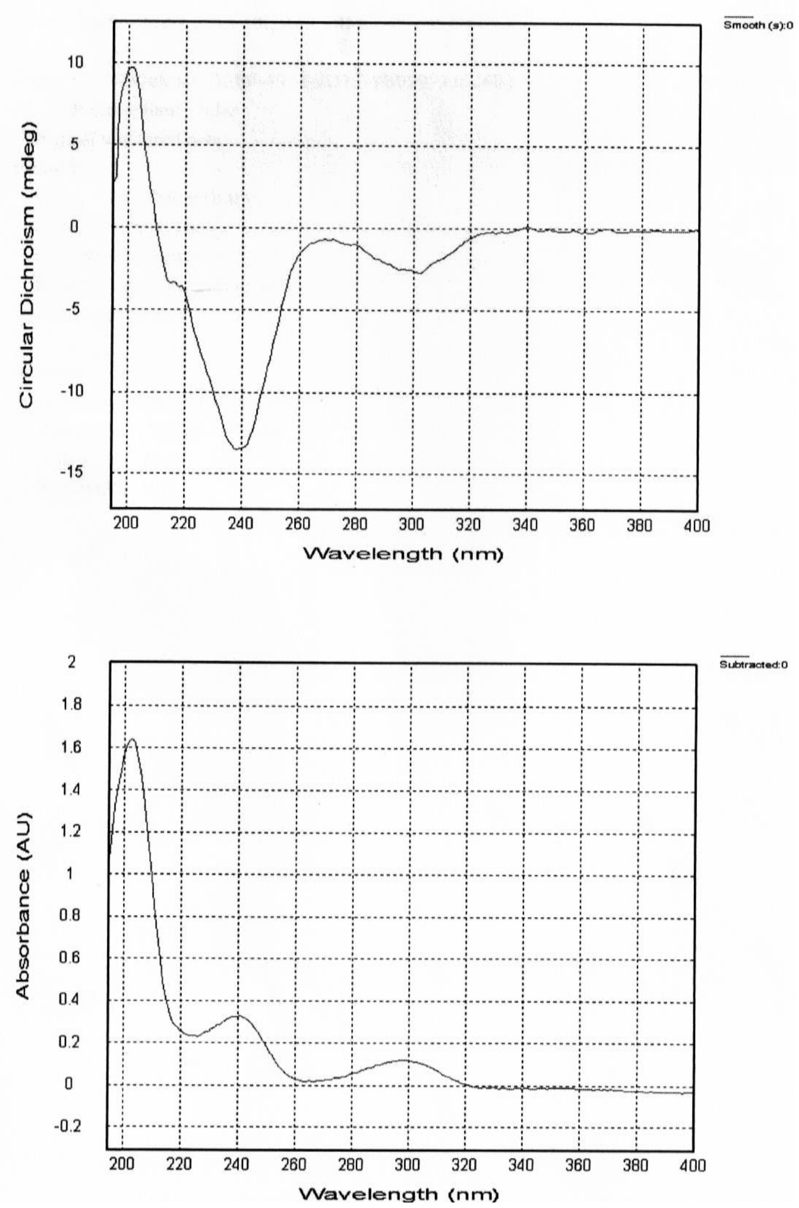

Fig. 17S CD spectrum of Alstoniascholarine M (**2**)

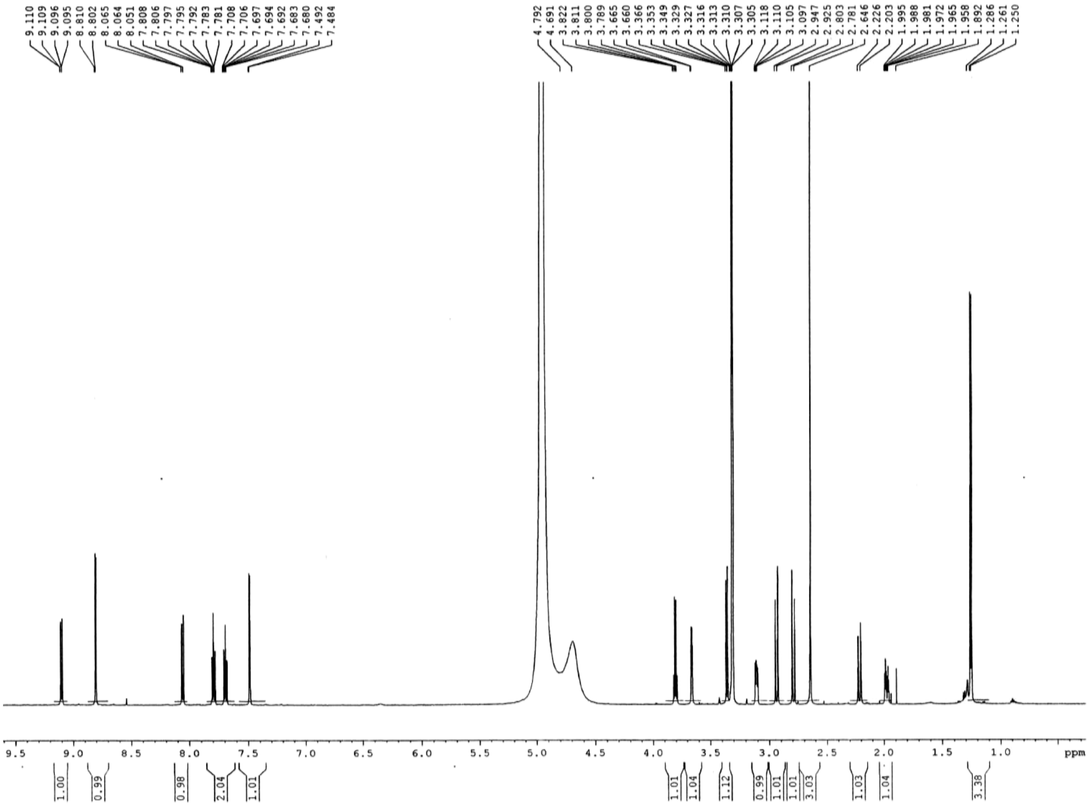

Fig. 18S ^{1}H NMR spectrum of Alstoniascholarine N (**3**) recorded at 600 MHz in CD_3OD

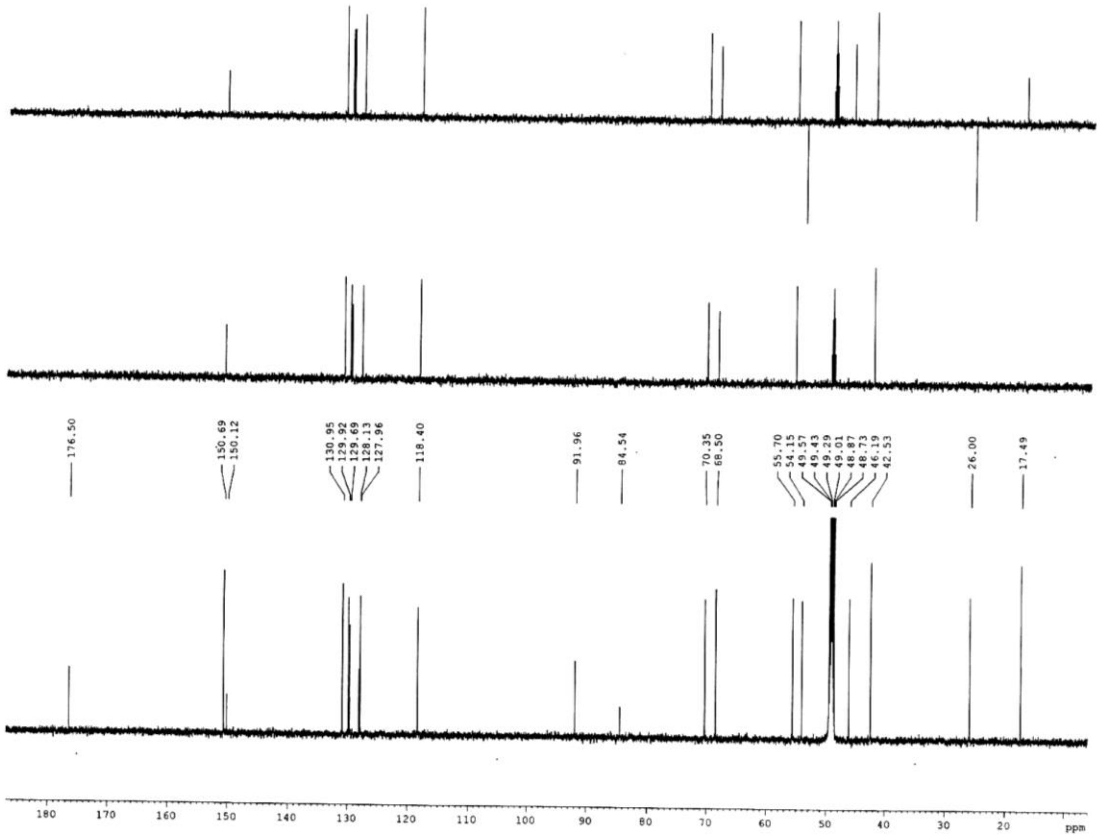

Fig. 19S ^{13}C NMR spectrum of Alstoniascholarine N (**3**) recorded at 150 MHz in CD_3OD

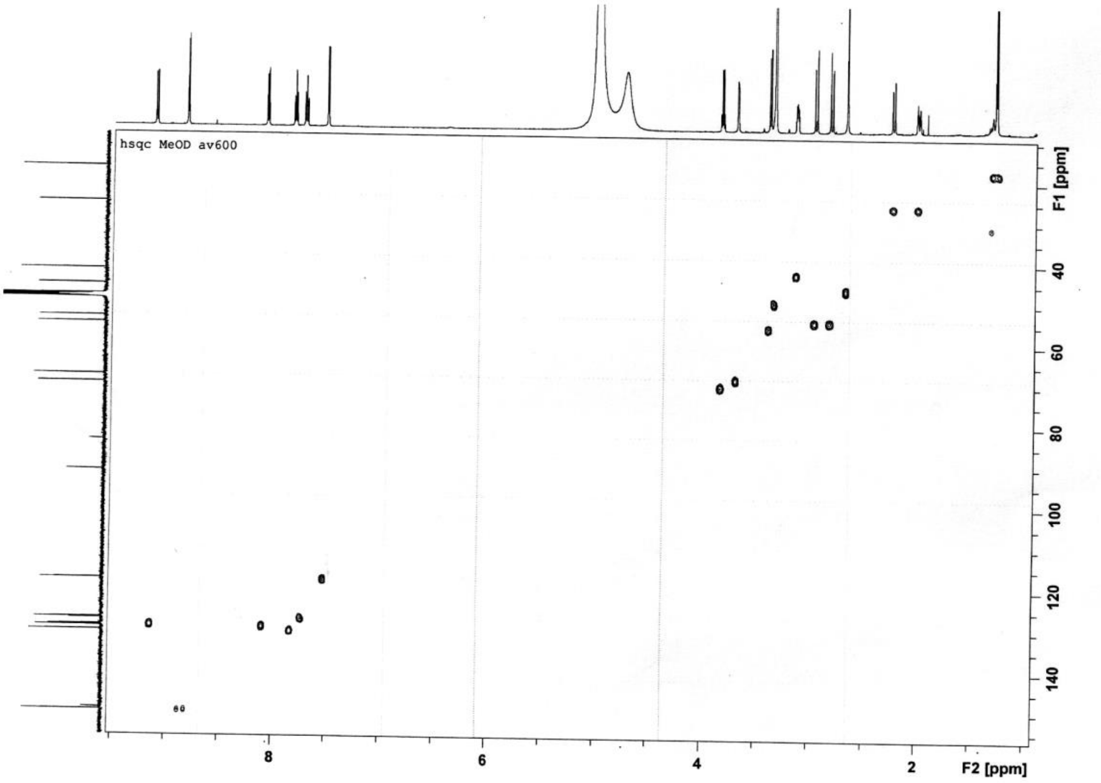

Fig. 20S. HSQC spectrum of Alstoniascholarine N (**3**)

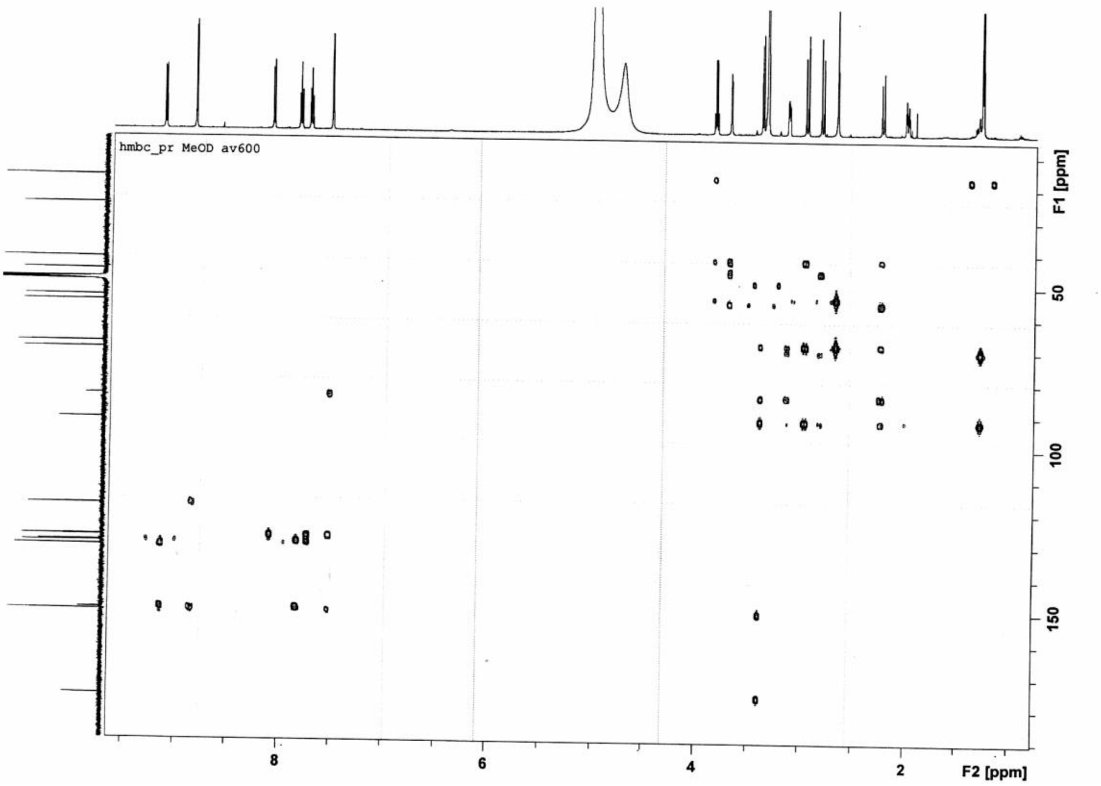

Fig. 21S HMBC spectrum of Alstoniascholarine N (**3**)

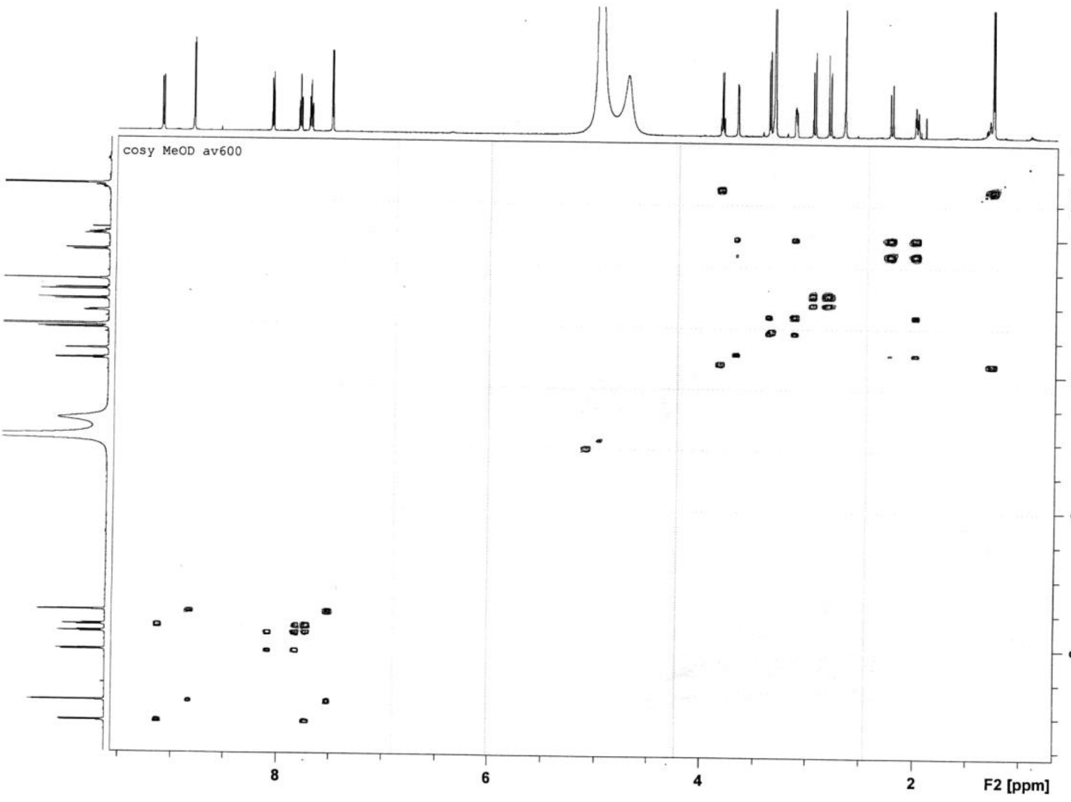

Fig. 22S ^{1}H-^{1}H COSY spectrum of Alstoniascholarine N (**3**)

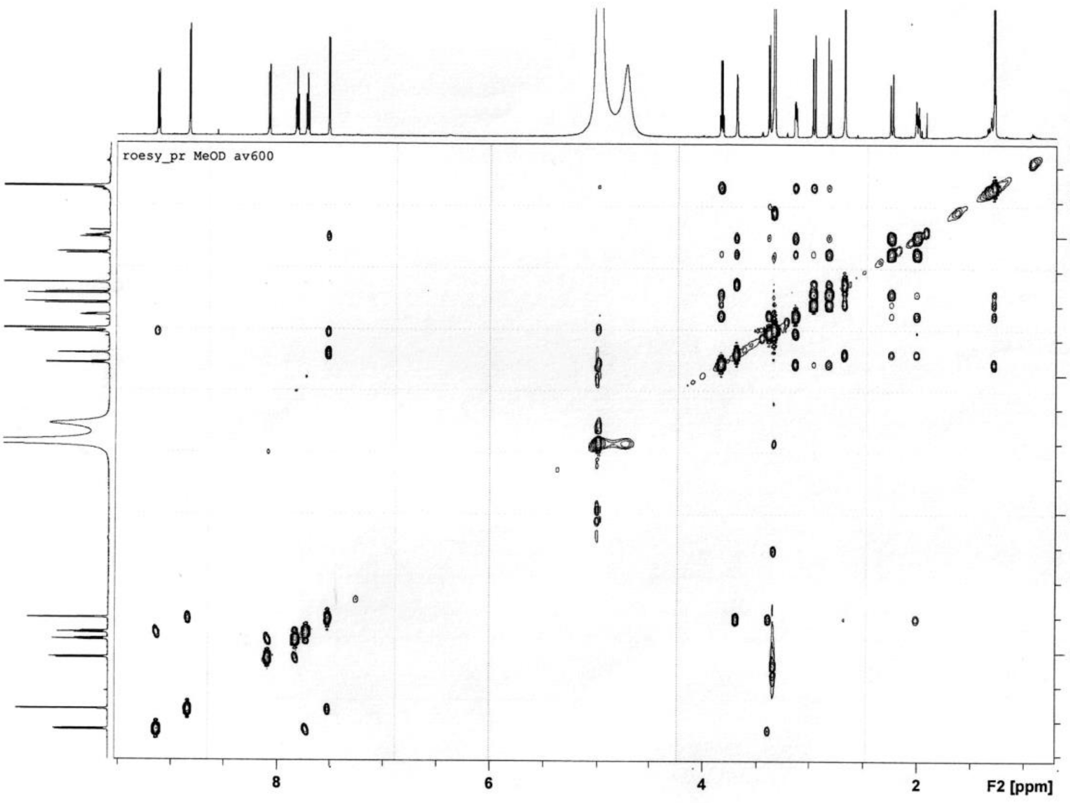

Fig. 23S ROESY spectrum of Alstoniascholarine N (**3**)

Elemental Composition Report Page 1

Single Mass Analysis
Tolerance = 10.0 PPM / DBE: min = -10.0, max = 120.0
Selected filters: None

Monoisotopic Mass, Odd and Even Electron Ions
16 formula(e) evaluated with 1 results within limits (up to 51 closest results for each mass)
Elements Used:
C: 0-200 H: 0-400 N: 2-2 O: 3-5

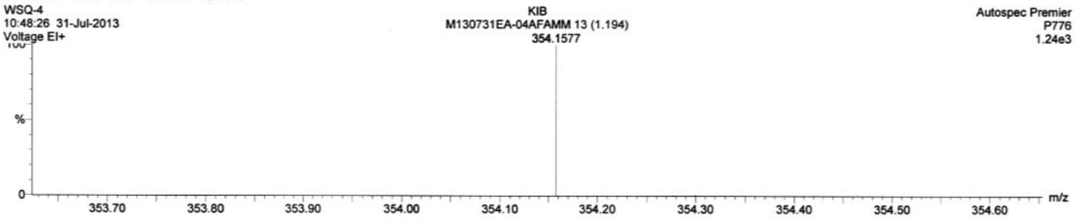

Minimum:				-10.0		
Maximum:		200.0	10.0	120.0		
Mass	Calc. Mass	mDa	PPM	DBE	i-FIT	Formula
354.1577	354.1580	-0.3	-0.8	11.0	5546630.5	C20 H22 N2 O4

Fig. 24S HREIMS spectrum of Alstoniascholarine N (**3**)

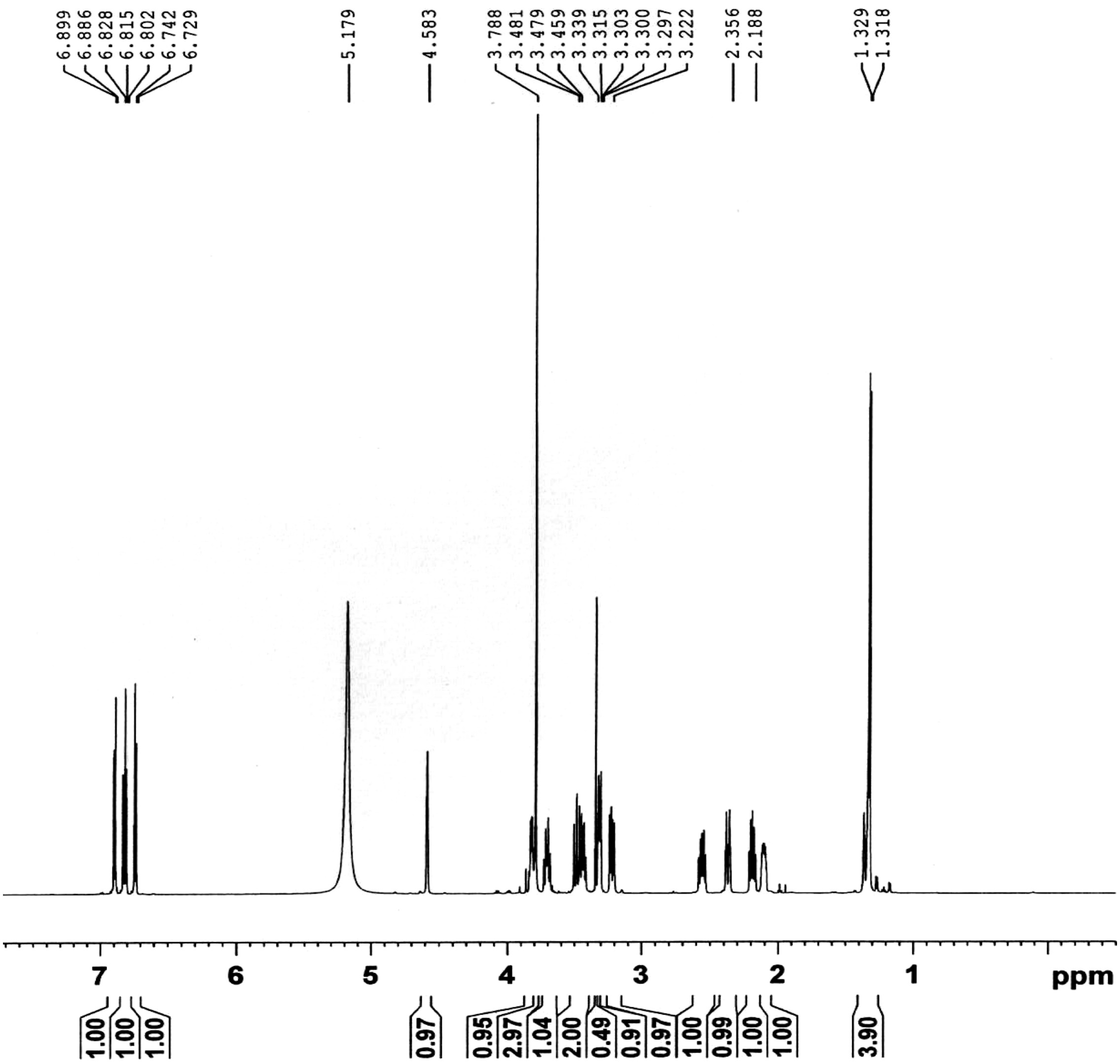

Fig. 25S ^{1}H NMR spectrum of Alstoniascholarine O (**4**) recorded at 600 MHz in CD_3OD

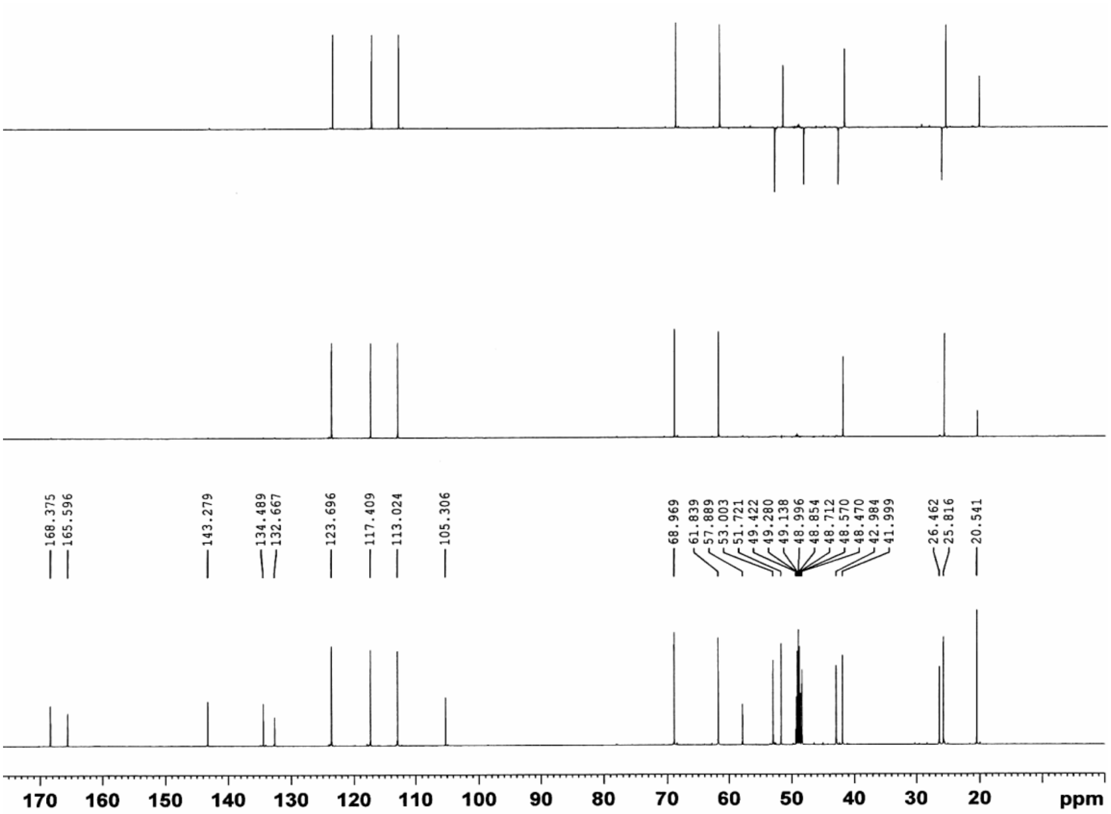

Fig. 26S ^{13}C NMR spectrum of Alstoniascholarine O (**4**) recorded at 150 MHz in CD_3OD

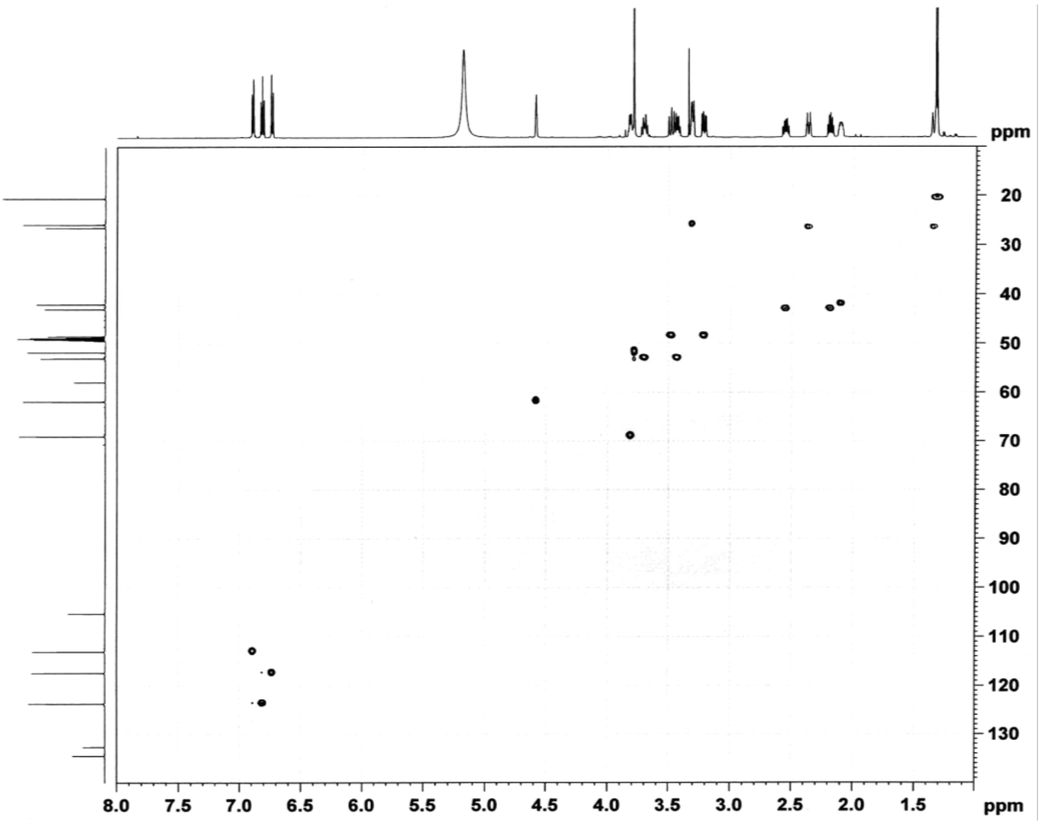

Fig. 27S HSQC spectrum of Alstoniascholarine O (**4**)

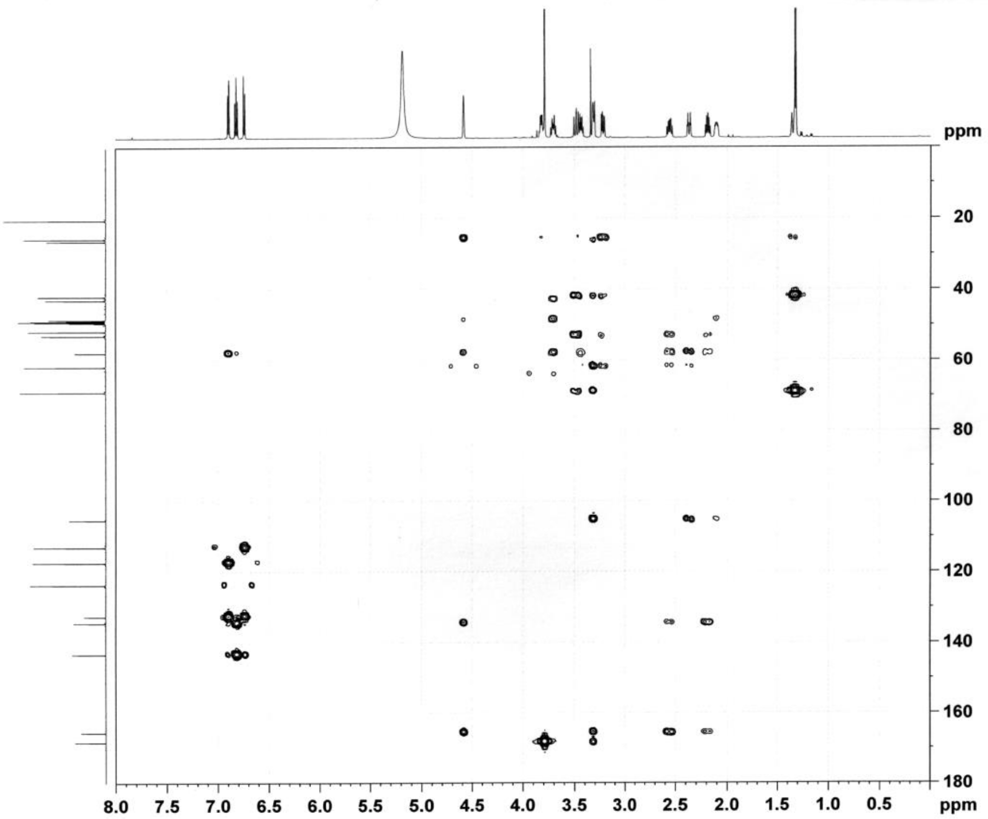

Fig. 28S HMBC spectrum of Alstoniascholarine O (**4**)

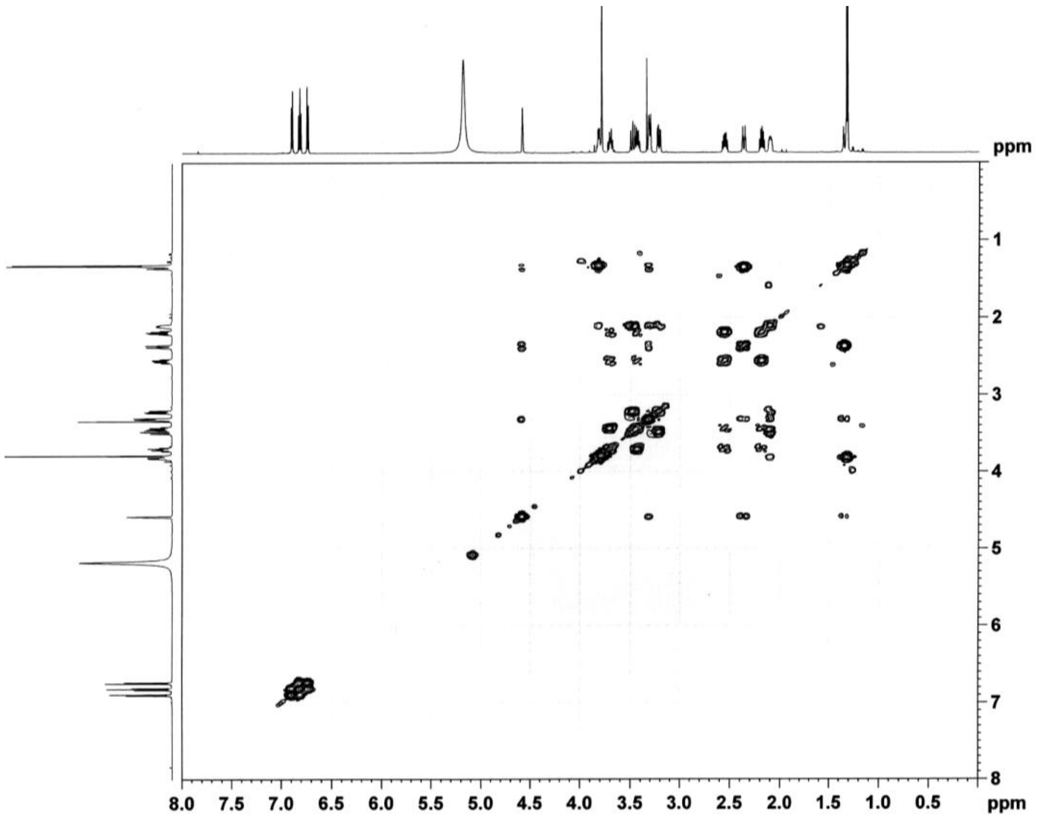

Fig. 29S ^{1}H-^{1}H COSY spectrum of Alstoniascholarine O (**4**)

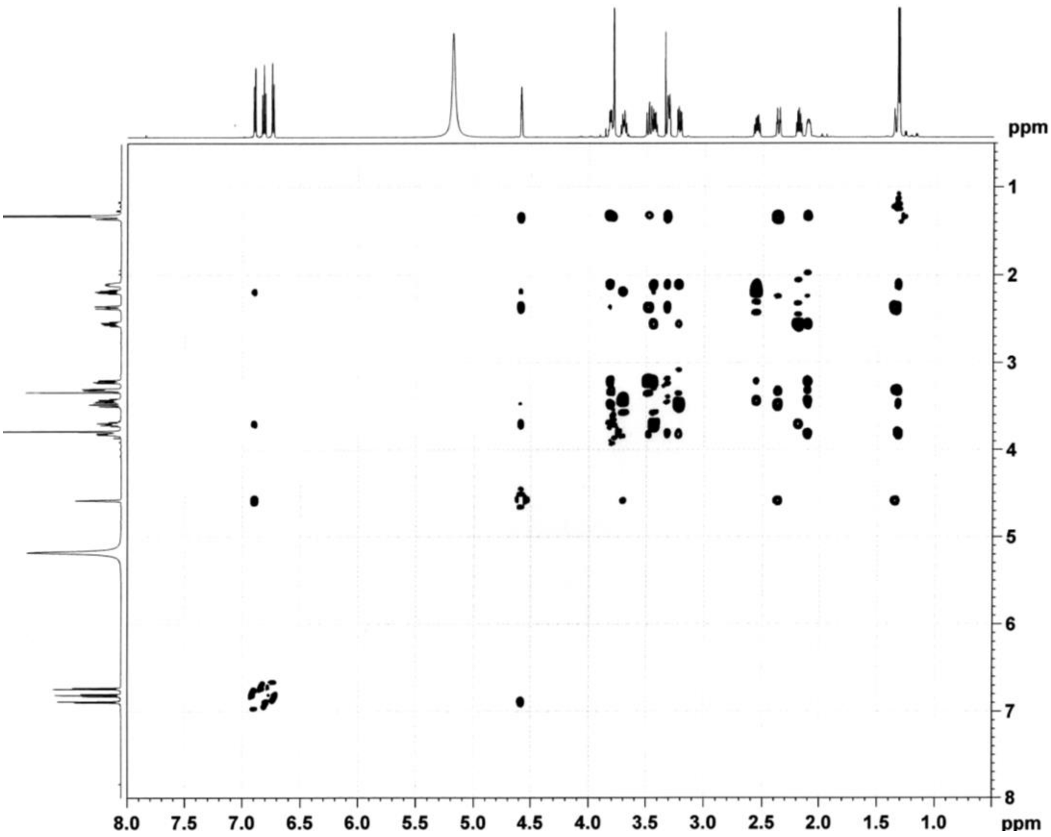

Fig. 30S ROESY spectrum of Alstoniascholarine O (**4**)

Qualitative Analysis Report

**Data Filename**	WSQ-26.d	**Sample Name**	WSQ-26
**Sample Type**	Sample	**Position**	P1-A4
**Instrument Name**	Instrument 1	**User Name**	
**Acq Method**	SIBU.m	**Acquired Time**	3/30/2015 4:06:48 PM
**IRM Calibration Status**	Success	**DA Method**	Default.m
**Comment**			

Sample Group **Info.**
Acquisition SW 6200 series TOF/6500 series
Version Q-TOF B.05.01 (B5125.2)

User Spectra

Fragmentor Voltage	Collision Energy	Ionization Mode
135	0	ESI

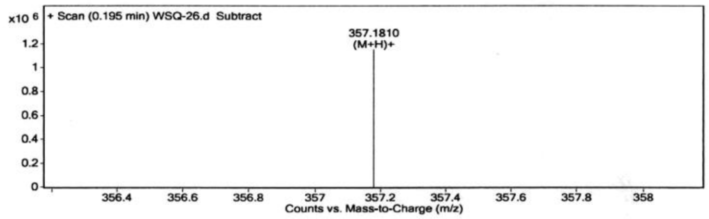

Peak List

m/z	z	Abund	Formula	Ion
140.0019	1	40749.42		
347.2211	1	8792.11		
357.181	1	1155960.5	C20 H24 N2 O4	(M+H)+
358.1845	1	234741.67	C20 H24 N2 O4	(M+H)+
359.1869	1	30762.07	C20 H24 N2 O4	(M+H)+
373.1763	1	18215.01		

Formula Calculator Element Limits

Element	Min	Max
C	3	60
H	0	120
O	0	30
N	0	10

Formula Calculator Results

Formula	CalculatedMass	CalculatedMz	Mz	Diff. (mDa)	Diff. (ppm)	DBE
C20 H24 N2 O4	356.1736	357.1809	357.1810	-0.2	-0.6	10.0000

Fig. 31S HRESIMS spectrum of Alstoniascholarine O (**4**)

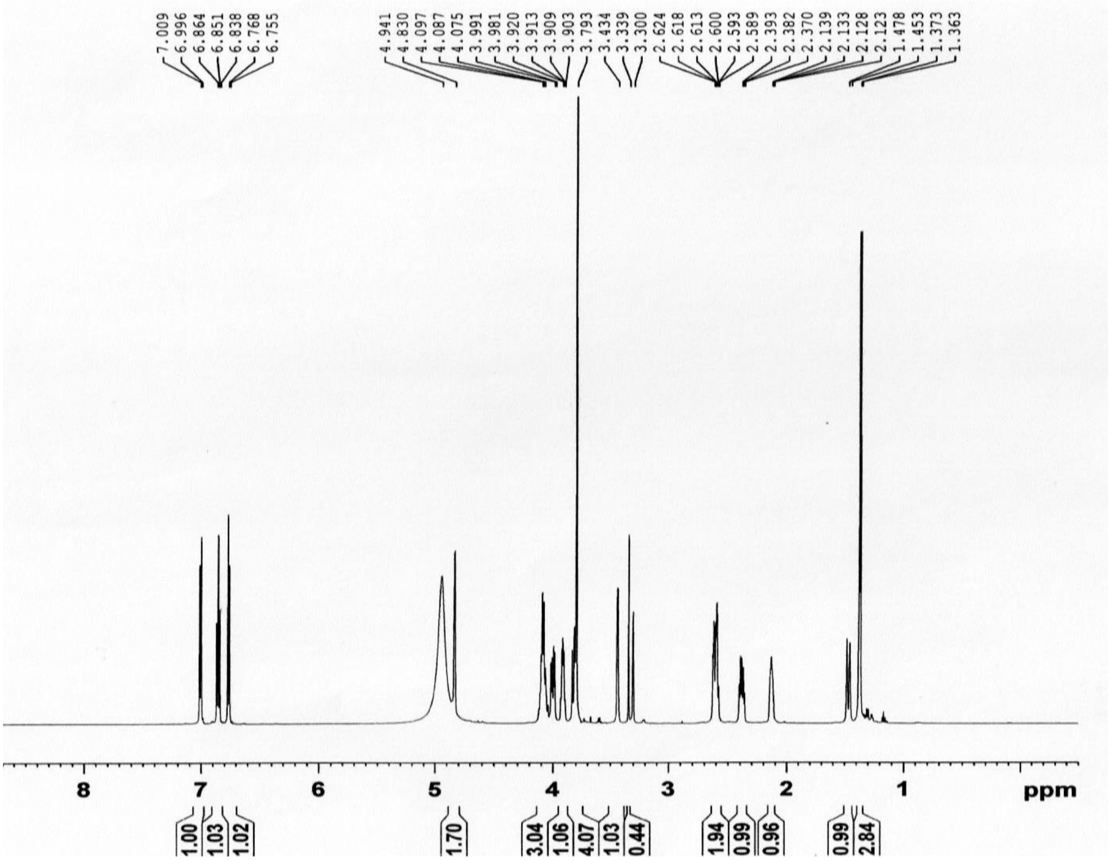

Fig. 32S ^{1}H NMR spectrum of Alstoniascholarine P (**5**) recorded at 600 MHz in CD_3OD

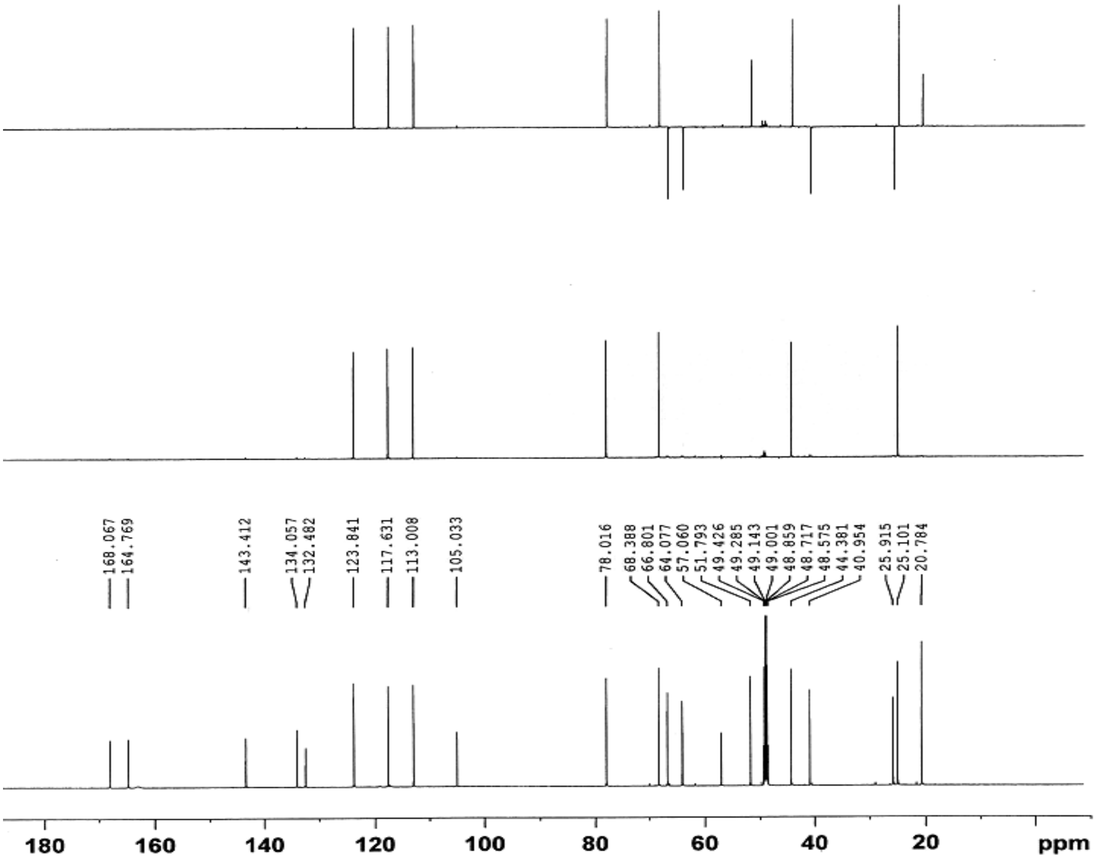

Fig. 33S ^{13}C NMR spectrum of Alstoniascholarine P (**5**) recorded at 150 MHz in CD_3OD

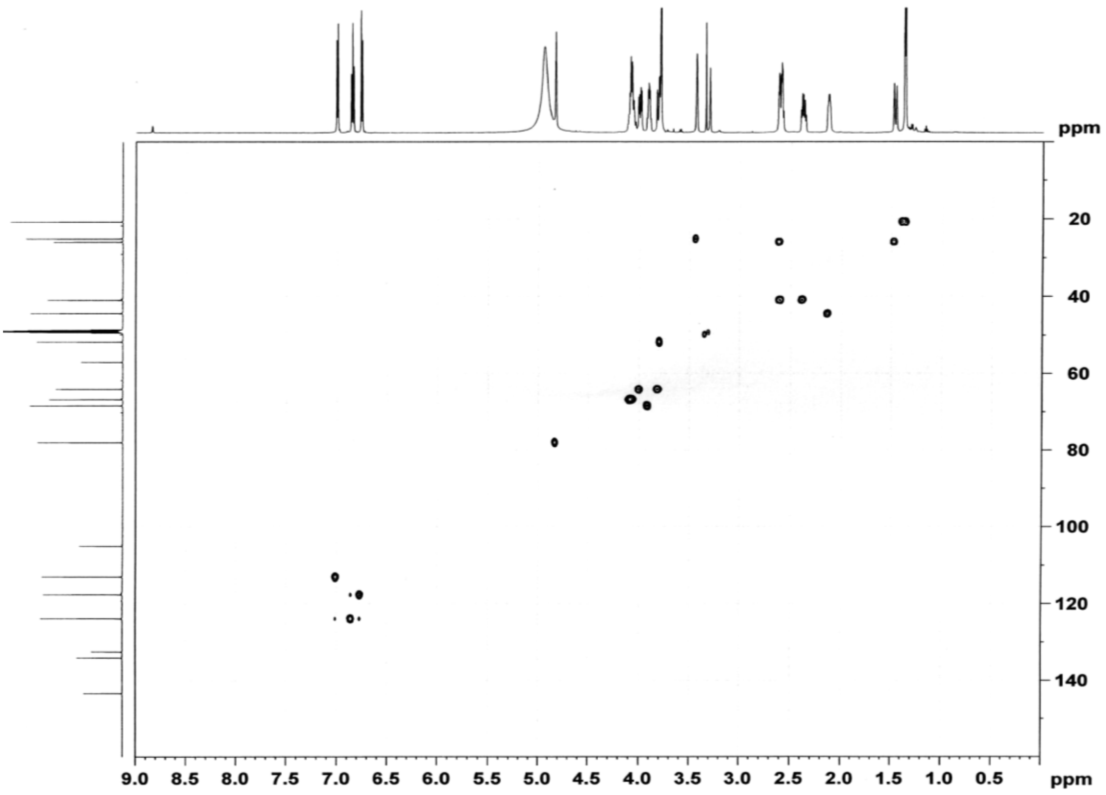

Fig. 34S HSQC spectrum of Alstoniascholarine P (**5**)

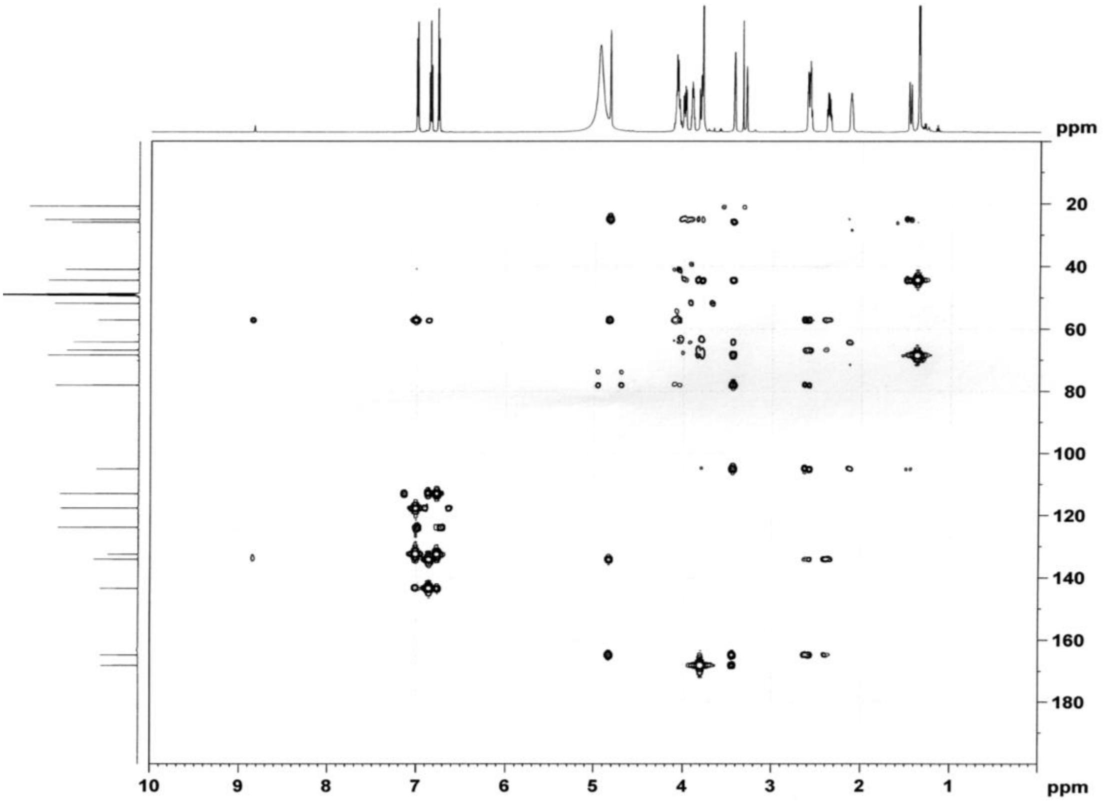

Fig. 35S HMBC spectrum of Alstoniascholarine P (**5**)

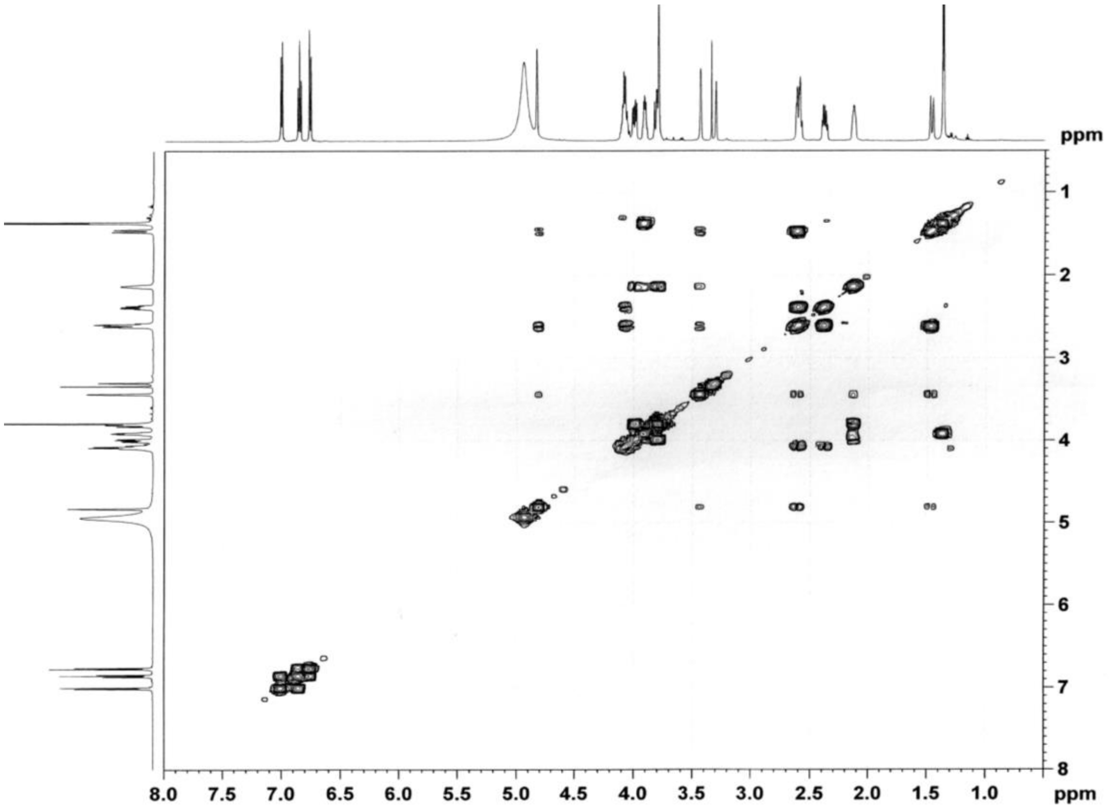

Fig. 36S ^{1}H-^{1}H COSY spectrum of Alstoniascholarine P (**5**)

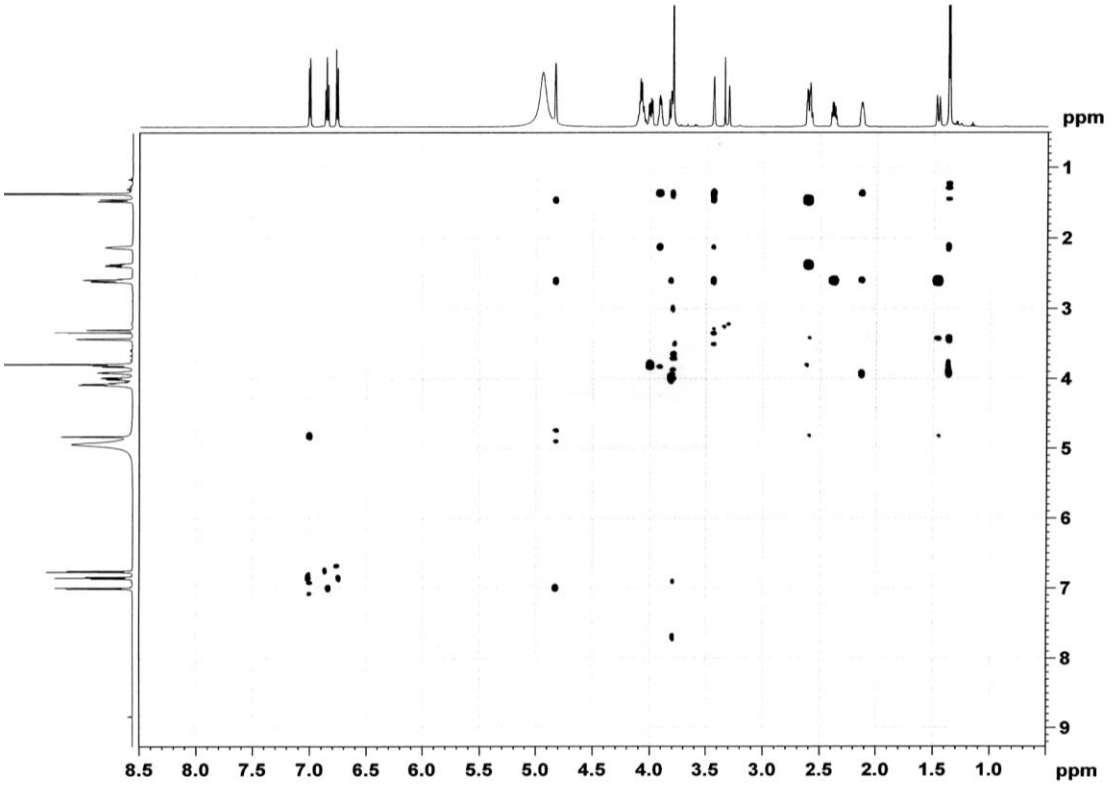

Fig. 37S ROESY spectrum of Alstoniascholarine P (**5**)

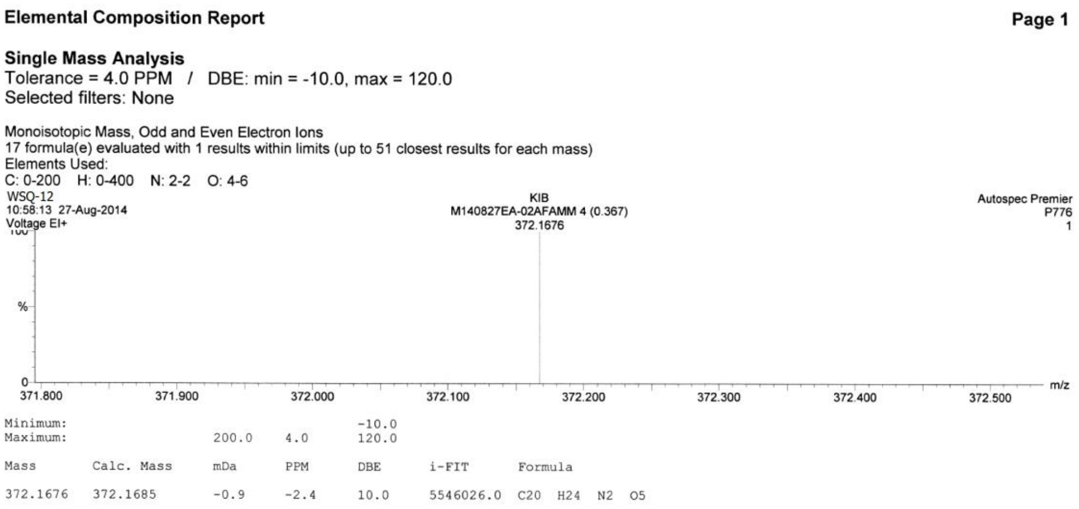

Fig. 38S HRESIMS spectrum of Alstoniascholarine P (**5**)

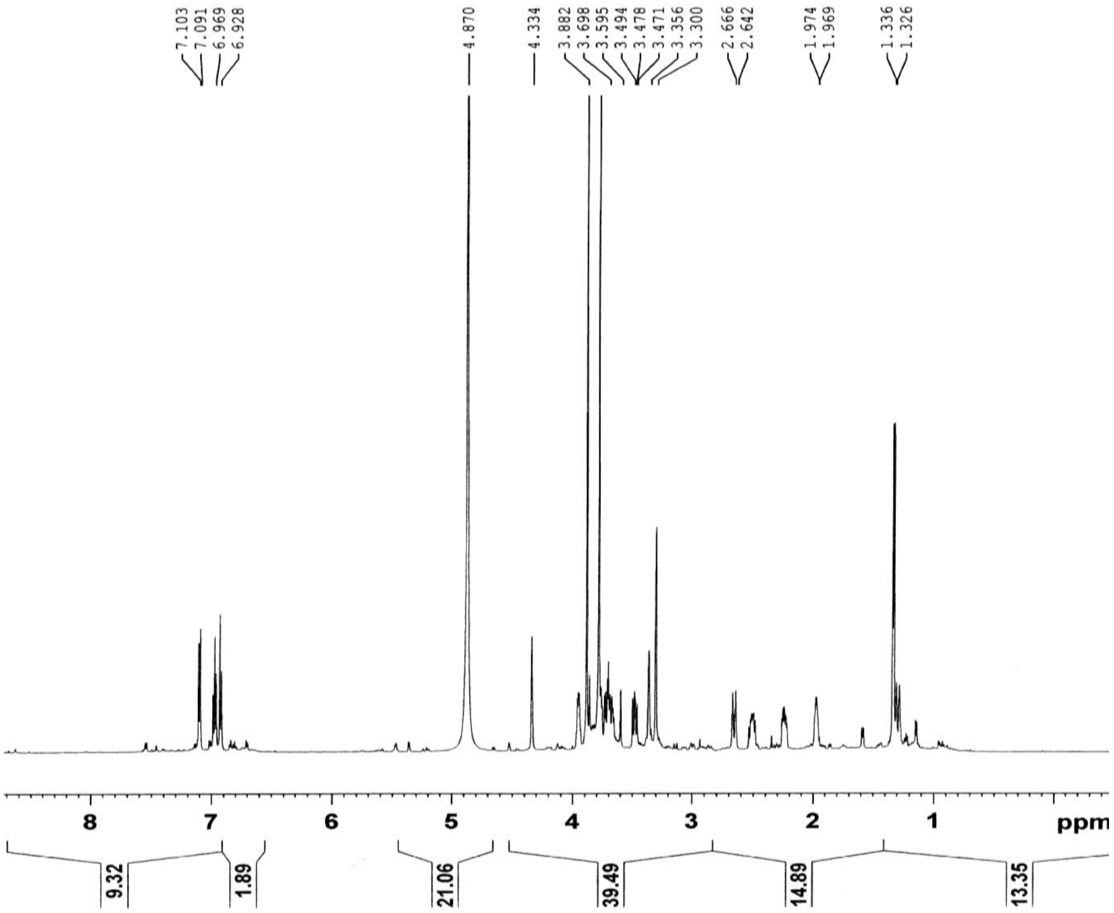

Fig. 39S ^{1}H NMR spectrum of Alstoniascholarine Q (**6**) recorded at 600 MHz in CD_3OD

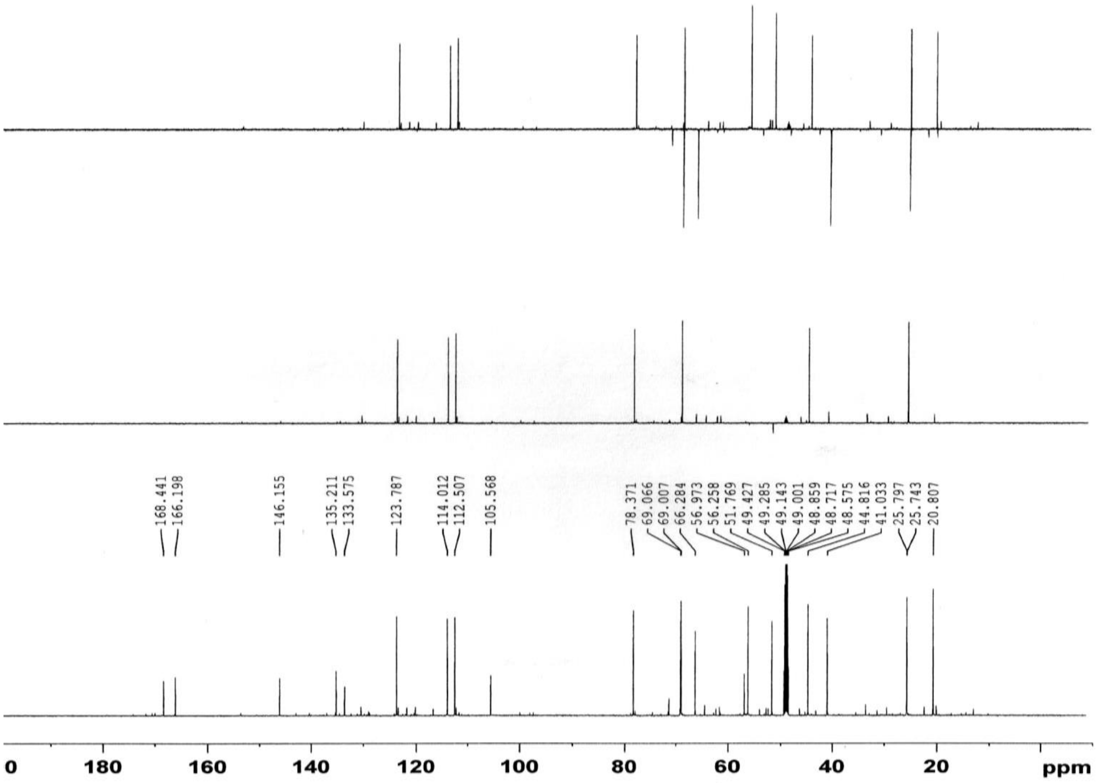

Fig. 40S ^{13}C NMR spectrum of Alstoniascholarine Q (**6**) recorded at 150 MHz in CD_3OD

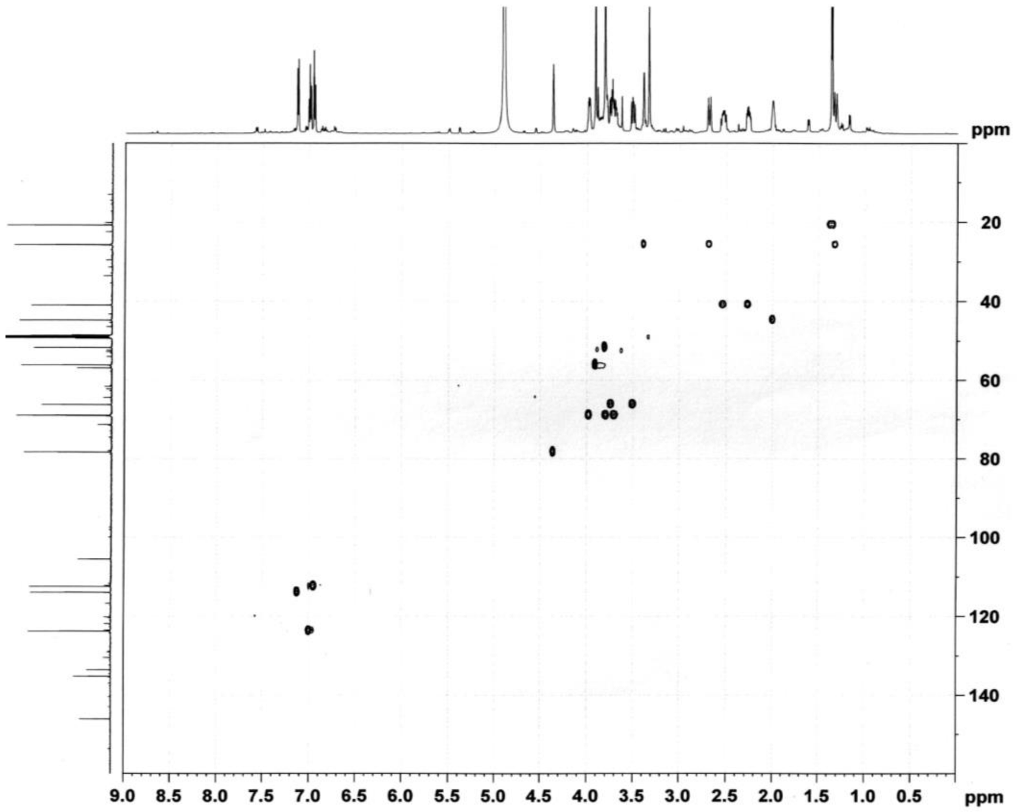

Fig. 41S HSQC spectrum of Alstoniascholarine Q (**6**)

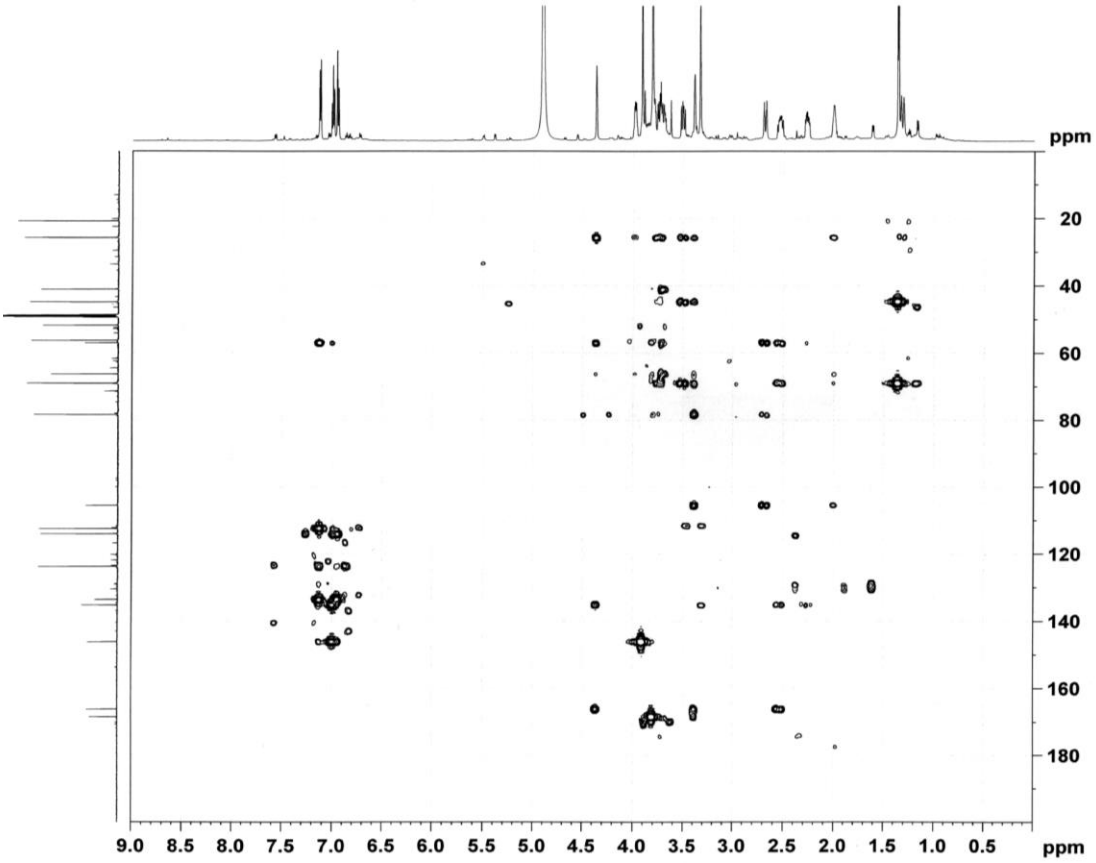

Fig. 42S HMBC spectrum of Alstoniascholarine Q (**6**)

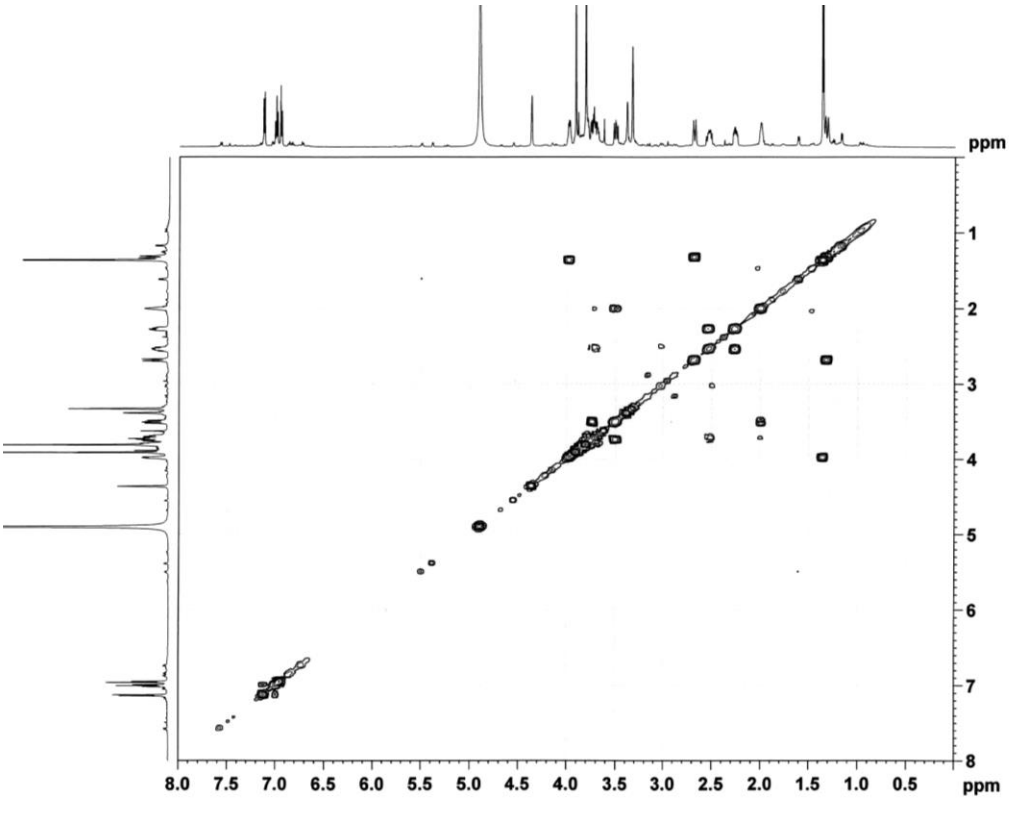

Fig. 43S ^{1}H-^{1}H COSY spectrum of Alstoniascholarine Q (**6**)

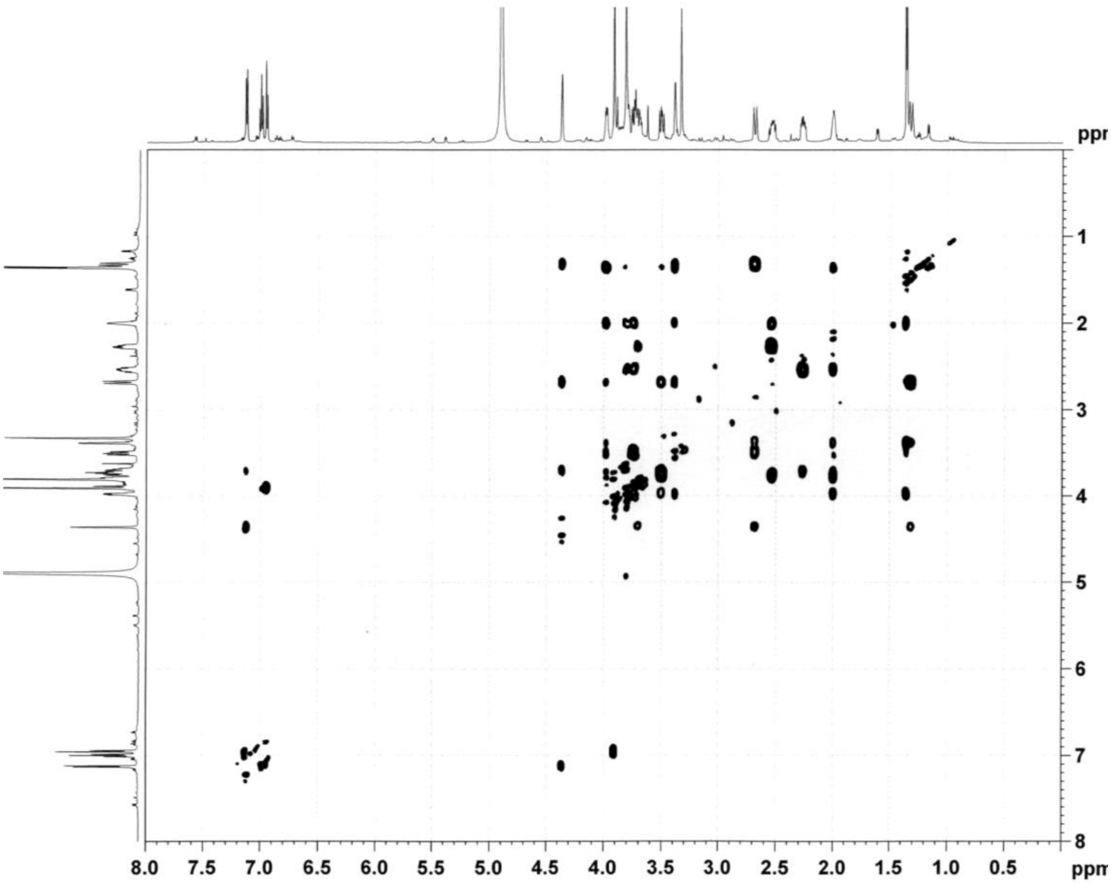

Fig. 44S ROESY spectrum of Alstoniascholarine Q (**6**)

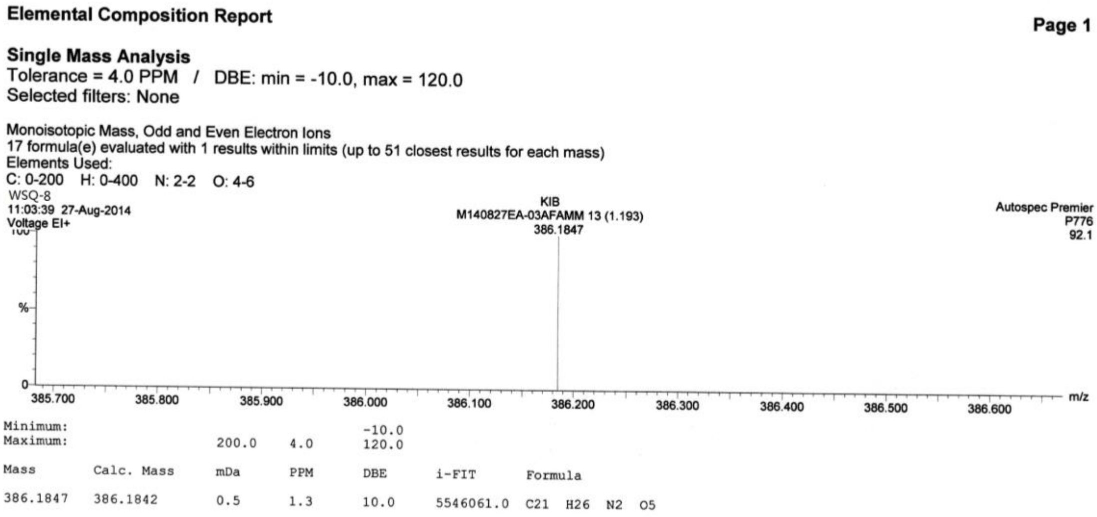

Fig. 45S HRESIMS spectrum of Alstoniascholarine Q (**6**)
